# Prebiotic Photochemical Coproduction of Purine Ribo- and Deoxyribonucleosides

**DOI:** 10.1021/jacs.1c07403

**Published:** 2021-09-01

**Authors:** Jianfeng Xu, Nicholas J. Green, David A. Russell, Ziwei Liu, John D. Sutherland

**Affiliations:** MRC Laboratory of Molecular Biology, Francis Crick Avenue, Cambridge Biomedical Campus, Cambridge CB2 0QH, U.K.

## Abstract

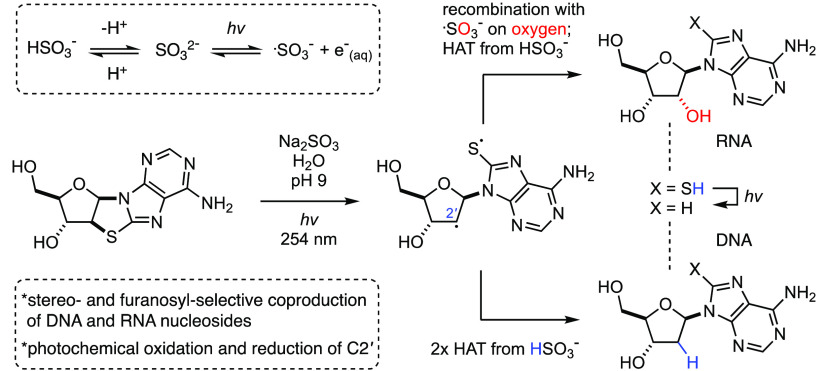

The hypothesis that life on Earth may have started with a heterogeneous nucleic acid genetic system including both RNA and DNA has attracted broad interest. The recent finding that two RNA subunits (cytidine, C, and uridine, U) and two DNA subunits (deoxyadenosine, dA, and deoxyinosine, dI) can be coproduced in the same reaction network, compatible with a consistent geological scenario, supports this theory. However, a prebiotically plausible synthesis of the missing units (purine ribonucleosides and pyrimidine deoxyribonucleosides) in a unified reaction network remains elusive. Herein, we disclose a strictly stereoselective and furanosyl-selective synthesis of purine ribonucleosides (adenosine, A, and inosine, I) and purine deoxynucleosides (dA and dI), alongside one another, via a key photochemical reaction of thioanhydroadenosine with sulfite in alkaline solution (pH 8–10). Mechanistic studies suggest an unexpected recombination of sulfite and nucleoside alkyl radicals underpins the formation of the ribo C2′–O bond. The coproduction of A, I, dA, and dI from a common intermediate, and under conditions likely to have prevailed in at least some primordial locales, is suggestive of the potential coexistence of RNA and DNA building blocks at the dawn of life.

The composition of Earth’s first genetic polymer has long been the subject of intense research.^1−3^ Contrary to the common “RNA world” hypothesis, the “R/DNA world” theory posits that the first genetic system might have comprised both RNA and DNA nucleotides.^4−6^ This scenario potentially circumvents the postulated “genetic takeover” of homogeneous DNA from RNA as the genetic information carrier in the RNA world,^7^ replacing it with a divergence of RNA and DNA subcomponents into more specialized roles. Recent developments in the prebiotic synthesis of purine deoxyribonucleosides (dA **1** and dI **2**)^8,9^ from *ribo*-aminooxazoline (RAO **3**, Scheme 1), a common intermediate in the synthesis of pyrimidine ribonucleosides (C **4** and U **5**),^10^ support the notion that RNA and DNA building blocks could have coexisted before life’s emergence. However, existing prebiotic syntheses of purine ribonucleosides^11−14^ depend on chemically and enantiomerically pure sugar starting materials unlikely to have existed on primordial Earth (e.g., ribose)^15^ or produce low yields of biologically relevant nucleosides among a plethora of undesirable isomers and congeners. Hence, a robust, stereoselective and furanosyl-selective synthesis of purine ribonucleosides remains an attractive goal of prebiotic synthesis. Because of its properties as a conglomerate, RAO **3**, which crystallizes enantiopure from solutions of minimally enantioenriched carbohydrates,^16,17^ is of interest as a common precursor to nucleosides.

**Scheme 1:**
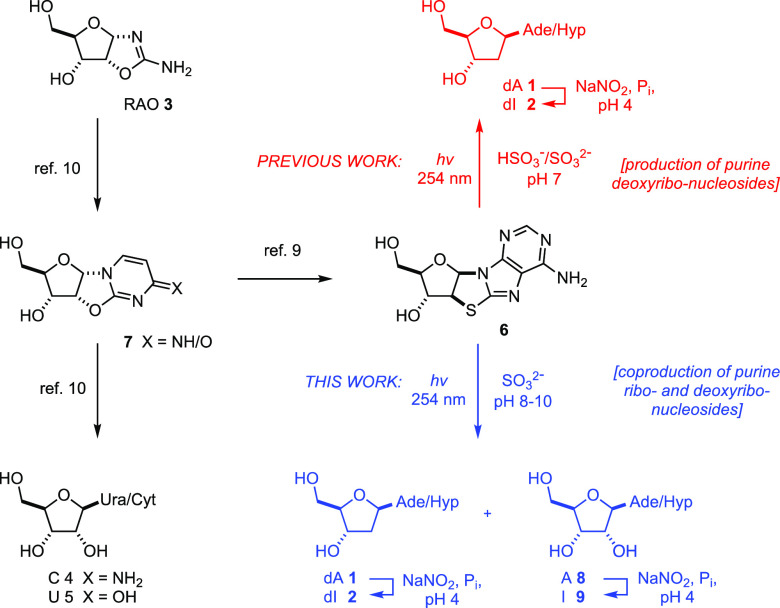
Synthesis of Purine Deoxyribonucleosides and Pyrimidine Ribonucleosides in a Unified Reaction Network RAO is a common precursor in previous works^9,10^ and the coproduction of purine ribo- and deoxyribonucleosides presented herein. Ade = *N*^9^-adeninyl; Hyp = *N*^9^-hypoxanthinyl; Ura = *N*^1^-uracilyl; Cyt = *N*^1^-cytosinyl; P_i_ = NaH_2_PO_4_.

Previously, we described a potentially prebiotic synthesis of deoxyadenosine **1** via photochemical reduction of thioanhydroadenosine **6** with sodium bisufite or hydrogen sulfide at pH 7. Thioanhydroadenosine **6** was furnished by tethered glycosylation with anhydropyrimidines **7**, derived from enantiopure RAO **3**.^9^ Our synthesis of dA **1** was ultimately completely selective for the canonical stereochemistry, regiochemistry, and furanosyl isomer of dA and thus constituted an advance over previous glycosylation strategies.^11,12,14^ However, recent studies suggest that alkaline lakes were likely common on primitive Earth and may have facilitated concentration of atmospheric HCN, CO_2_, and SO_2_ into groundwater.^18,19^ These findings prompted us to revisit the photochemistry of thioanhydroadenosine **6** with sulfite (SO_3_^2–^) at alkaline pH (8–10). Our investigations have led to the discovery of an equally viable potentially prebiotic route to purine ribonucleosides (**8**, **9**) alongside their deoxyribose congeners (**1**, **2**) via an unexpected novel mechanism.

## Alkaline Photochemical Reactivity

. When we irradiated (mercury lamp, 254 nm principal emission) a solution of thioanhydroadenosine **6** and sodium sulfite (4.5 equiv) at pH 9, eight nucleoside products were observed (Table 1). In addition to deoxyadenosine **1** and its 8-mercapto derivative **10**, which were major products obtained in our previous study at pH 7,^9^ the ribonucleosides adenosine **8** and 8-mercaptoadenosine **11** were identified by NMR spectroscopy and spiking with authentic samples (Figures S25 and S26). The remaining four products were isolated by preparative HPLC and characterized by NMR spectroscopy as adenosine-2′-α and adenosine-2′-β-sulfonates **12** and **13**, respectively, and their corresponding 8-mercapto derivatives **14** and **15** (Figures S1–S24). All of the 8-mercaptonucleosides (**10**, **11**, **14**, and **15**) were gradually converted to their desulfurized structures (**1**, **8**, **12**, and **13**, respectively) after further irradiation (12–24 h, Figures S25c and S27–S29). The yields of nucleosides obtained at various pH values are summarized in Table 1. The optimum combined yield for ribonucleosides **8** and **11** was 15% at pH 9 after irradiation for 5 h, which also provided deoxynucleosides **1** and **10** in 43% combined yield (entry 3, Table 1).

**Table 1:**
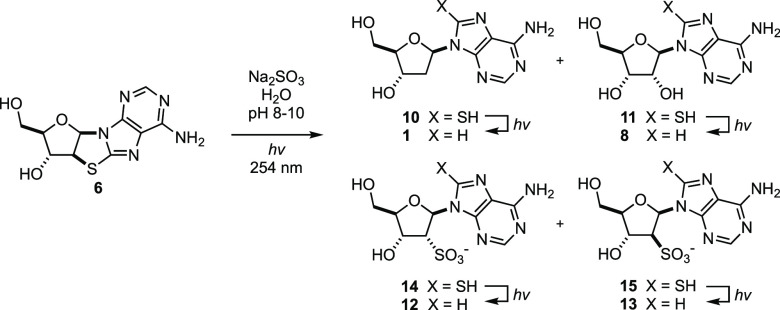
Summary of the Yields of Different Products Obtained Following Irradiation of Thioanhydroadenosine **6** with Sulfite at pH 7–10 for 5 h

		combined yields of products^a^ (%)			
entry	pH	**1** + **10**	**8** + **11**	**12** + **14**	**13** + **15**
1^b^	7	75			
2	8	56	10	14	13
3	9	43	15	20	18
4	10	32	12	17	15

a Yields are based on relative integration of the signals in ^1^H NMR spectra compared to an internal standard (pentaerythritol).

b Yields as reported in ref (9).

## Conversion to Purine Alphabet

**Scheme 2:**
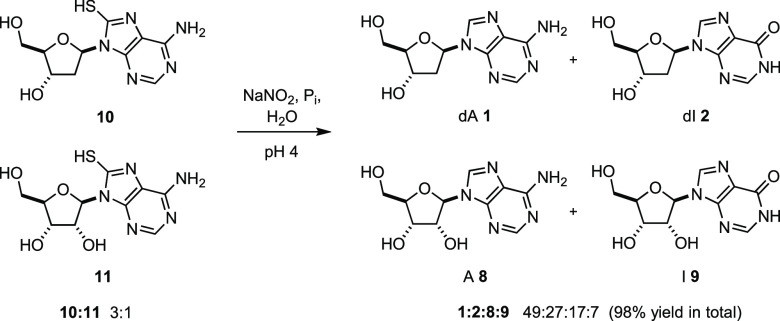
Nitrosative Deamination and Desulfurization of a 3:1 Mixture of **10** and **11** Gives a Mixture of dA, dI, A, and I (P_i_ = NaH_2_PO_4_)

. Nitrosative desulfurization and partial deamination were previously shown to convert deoxyadenosine **1** and its 8-mercapto precursor **10** to a mixture of dA and dI.^9^ With a mixture of ribo- and deoxyribonucleosides **10** and **11** in hand, we evaluated their reactivity in this context to see if we might additionally generate inosine **9**, a potential surrogate for guanosine in the primordial genetic alphabet. Inosine functions in nonenzymatic RNA replication systems without loss of rate or fidelity compared to guanosine.^20^ We therefore subjected a 3:1 mixture of **10** and **11** (the distribution of these products obtained after irradiating **6** for 2 h with sulfite at pH 9) to nitrosation at pH 4.^21,22^ After 12 days at room temperature in the presence of sodium nitrite (10 equiv) and sodium phosphate, initially at pH 4, A **8**, I **9**, dA **1**, and dI **2** were obtained in 17%, 6%, 48%, and 27% yield, respectively (Scheme 2 and Figure S38). Thus, both the ribo- and deoxyribofuranosides of adenine and hypoxanthine (**8**, **1**, **9**, and **2**) are available concomitantly, raising the possibility that these nucleosides formed components of a primordial genetic alphabet, ultimately surviving the test of evolution to varying degrees. The potential role or attrition of sulfonates **12**–**15**, in prebiotic oligomerization and replication processes, is currently under investigation.

## Molecular Mechanism

**Scheme 3:**
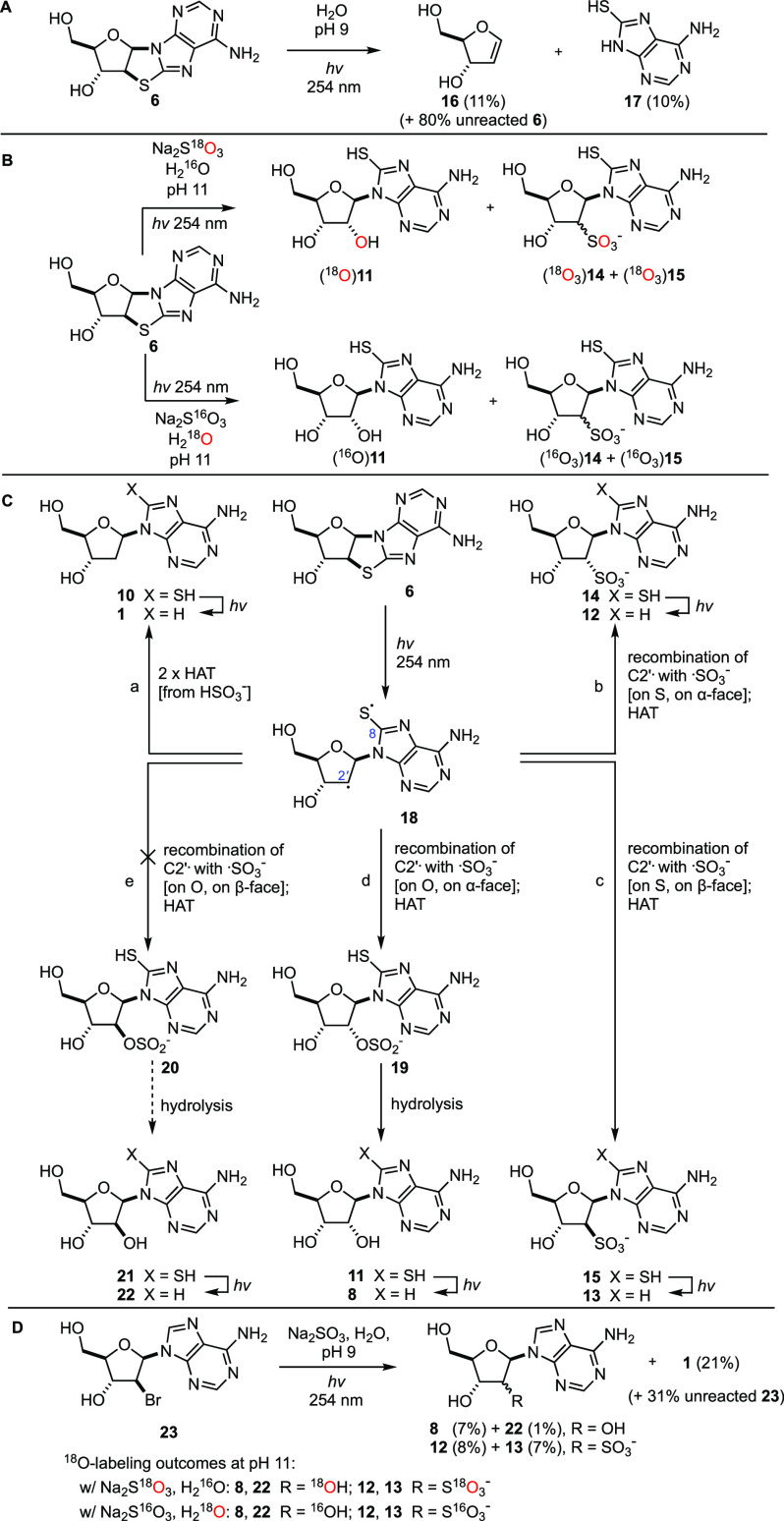
Proposed Mechanism for the Coproduction of Purine Ribonucleosides and Deoxyribonucleosides in the Photochemical Reaction of **6** with Sulfite at pH 8–10 (A) In the absence of sulfite, **6** decomposes to glycal **16**. (B) Positive and negative ^18^O-labeling experiments (top and bottom, respectively). Analysis by LCMS revealed the source of the 2′-oxygen of adenosine precursor **11** to be the sulfite ion, not water or molecular oxygen. (C) The proposed mechanistic pathways for alkaline sulfite-mediated photochemical processing of thioanhydroadenosine. Path A: double hydrogen atom transfer furnishes deoxynucleosides (**1**, **10**). Path B: radical recombination between the sulfite radical at the sulfur atom of sulfite and the α-face of the C2′ radical of **18**, then HAT, affords α-sulfonates (**12**, **14**). Path C: radical recombination between the sulfite radical at the oxygen atom of sulfite and the α-face of the C2′ radical of **18**, then HAT and rapid sulfite ester hydrolysis, affords ribonucleosides (**8**, **11**). Path D: no arabino-configured products (**21**, **22**)^27^ were observed. Path E: formation of β-sulfonates (**13**, **15**) by radical recombination between the sulfite radical at the sulfur atom of sulfite and the β-face of the C2′ radical of **18**. (D) Photochemical experiments using a model substrate **23** show a similar outcome with reduced stereoselectivity for C–O bond formation.

. Intrigued by the appearance of sulfonates and adenosine derivatives in the photochemical reaction, we probed the mechanism of these transformations. First, we verified that no reaction takes place without irradiation. Second, we performed the reaction with **6** (ε = 6319 M^–1^ cm^–1^) in the absence of sulfite (ε = 20 M^–1^ cm^–1^) (Scheme 3A) and observed ribal **16** and 8-mercaptoadenine **17** (11% and 10% yield, respectively, after 2 h, 80% unreacted starting material). Finally, we performed ^18^O-labeling experiments to determine the source of oxygen in the products (Scheme 3B). These reactions were performed at pH 11 and for shorter reaction times (25–60 min) to mitigate oxygen exchange between isotopically differentiated sulfite and water.^23−25^ Positive and negative labeling experiments demonstrate that the source of oxygen in **11** is sulfite. Together, these results exclude a hydrolytic mechanism for the formation of **11** and indicate that **11**, **14**, and **15** are formed by radical coupling between C2′ of a putative photochemically generated diradical intermediate **18** (Scheme 3C)^9^ and a sulfite radical, at either sulfur or oxygen, followed by net hydrogen atom transfer (HAT) to the respective C8S radical. Sulfonate **14** is generated by reaction on the α-face of **18** with sulfite through sulfur, then HAT (Scheme 3C, path b), and adenosine precursor **11** is generated by reaction on the α-face of **18** with sulfite through oxygen, then HAT, to form sulfite ester **19** which is rapidly hydrolyzed^26^ to **11** (Scheme 3C, path d). Photochemical desulfurization of all 8-mercaptopurine intermediates^27^ (**10**, **11**, **14**, and **15**) and production of deoxyadenosine **1** (Scheme 3C, path a)^9^ are likely to take place via previously reported mechanisms.

No arabino-configured oxygenated products (**21**, **22**)^27^ were observed (Scheme 3C, path e), even though **22** is stable under the reaction conditions (Figure S30) and so could in principle accumulate. Thus, radical coupling between C2′ and sulfite O is highly stereoselective, in contrast to coupling at sulfite S, which provides the α- and β-sulfonates **14** and **15**. To investigate this stereoselectivity, we synthesized 2′-deoxy-β-bromoadenosine **23**,^28^ so we could generate a model putative radical at C2′ by reductive photochemical cleavage of the C2′–Br bond.^29^ When we submitted **23** to UV irradiation in the presence of sulfite at pH 9 (Scheme 3D), we observed the expected products, deoxyadenosine **1**, adenosine **8**, and sulfonates **12** and **13**, but also arabino-adenosine **22** (Scheme 3D). Labeling experiments indicate the same mechanism is operating. The α/β ratio of sulfonate stereoisomers was ∼53:47 (similar to the reaction with thioanhydroadenosine **6**, ∼50:50) and the ratio of ribo- to arabino-adenosine (**8**:**22**) was ∼85:15. We therefore conclude that the high stereoselectivity for radical recombination of sulfite at oxygen (for both **6** and **23**) is enforced mostly by the substrate structure of adenosine and enhanced by the presence of the 8-mercapto group in **6**/**18**. This is likely due to steric shielding of the β-face of the C2′ radical intermediates by the nucleobase, which is increased by presence of the 8-mercapto group in **18**. C–O bond formation is substantially more affected than C–S bond formation by this shielding because of the corresponding shorter developing bond length in the respective transition states (an average C–O bond is ca. 1.4 Å while an average C–S bond is ca. 1.8 Å^30^). Accordingly, the 2′-α/β selectivity for ribo-adenosine over arabino-adenosine is high, but there is little 2′-α/β selectivity in the formation of sulfonates.

Thus, we propose that the coproduction of deoxyadenosine and adenosine in the alkaline sulfite-promoted photochemical reaction of thioanhydroadenosine **6** is mediated by both reductive and oxidative transformations of putative photochemically generated diradical intermediate **18**. In contrast to the exclusive reduction of **6** observed at pH 7 (entry 1, Table 1; Scheme 3C, path a),^9^ at alkaline pH (8–10) the ratio [SO_3_^2–^]:[HSO_3_^–^] is higher,^31^ favoring coupling of photochemically generated^32^ radicals over double HAT from HSO_3_^–^ (Scheme 3C, path a). This proposed mechanism is consistent with the higher proportion of sulfonates and ribonucleosides, and the lower proportion of deoxynucleoside products, produced at higher pH (entries 2–4, Table 1). Although sulfite radicals possess radical character at both S and O atoms,^33^ reactions predominate at S, and this is to the best of our knowledge the first reported radical coupling reaction between an alkyl radical and sulfite radical at oxygen. The most closely related example we found was the suggestion by Kolker and Lapworth^34^ that alongside reaction at S to form sulfonates, sulfite radicals may also react with some alkenes at O to form sulfites. We propose that the absence of the usually complete selectivity for radical sulfite reaction at S in our case is due to the highly reactive nature of the two radicals undergoing combination.

## Summary

. We show that coproduction of a purine R/DNA alphabet of nucleosides A, I, dA, and dI is enabled by a novel photochemical reaction of thioanhydroadenosine **6** with sulfite at pH 8–10. Mechanistic studies suggest the putative diradical **18** generated by photolysis of **6** undergoes either reduction or oxidation, with respect to the C2′ radical, to varying extents depending on pH. Oxidation appears to proceed by the surprisingly substantial combination of an alkyl radical with a sulfite radical at oxygen. Because sulfite and UV light are likely to have been commonplace in primordial environments,^35,36^ which would undoubtedly have varied in pH at locales or intervals in time,^18,19^ such prebiotic processing of thioanhydroadenosine **6** seems possible. Moreover, as **6** is derived from a common precursor used in the prebiotic synthesis of pyrimidine ribonucleosides (C and U), an extended genetic alphabet of RNA and DNA nucleosides (C, U, A, I, dA, and I) could have been available on early Earth via a unified chemical network and geochemical scenario. Finally, the key photochemical reaction mechanism proposed herein precludes the formation of nucleosides of noncanonical stereochemistry or sugar isomerism, consistent with the idea ultraviolet light not only provided energy essential for prebiotic chemistry but also enforced remarkable selectivity for biomolecules.^37^

## Supporting Information Available

General methods, synthetic procedures, and Figures S1–S50 (PDF)
