# Supplementary material for: Prebiotic Photochemical Coproduction of Purine Ribo- and Deoxyribonucleosides
Source: J Am Chem Soc. 2021 Sep 1;143(36):14482–6. doi: 10.1021/jacs.1c07403 (PMC8607323; doi:10.1021/jacs.1c07403)
Supplement: Supplementary file 1 — ja1c07403_si_001.pdf [file ja1c07403_si_001.pdf]

Prebiotic photochemical coproduction of purine ribo- and deoxyribo-nucleosides

Jianfeng Xu*, Nicholas J. Green, David A. Russell, Ziwei Liu and John D. Sutherland*

MRC Laboratory of Molecular Biology, Francis Crick Avenue, Cambridge Biomedical Campus, Cambridge, CB2 0QH, UK.

*Correspondence to: jxu@mrc-lmb.cam.ac.uk; johns@mrc-lmb.cam.ac.uk

This PDF file includes:

Materials and Methods
Table S1-S6
Supplementary Figures S1 – S50
References

Materials and Methods

General Methods

Reagents and deuterated solvents used for reactions were purchased from Sigma-Aldrich or Acros Organics and were used without further purification. All photochemical reactions were carried out in Norell Suprasil quartz NMR tubes purchased from Sigma-Aldrich using Hg lamps with principal emission at 254 nm in a Rayonet photochemical chamber reactor RPR-200, acquired from The Southern New England Ultraviolet Company. A Mettler Toledo SevenEasy pH Meter S20 was used to monitor the pH, and deoxygenation of solution was achieved by sparging anhydrous argon through the solution for 15-20 min. A preparative Varian Prostar HPLC System was used for the reverse phase high-pressure liquid chromatography (RP-HPLC) linked with an Atlantis T3 C18 Prep Column OBD 10 µm (19 × 250 mm). An Analytical HPLC was used to monitor the reaction, using a Thermofisher Ultimate 3000 UPLC and Waters Atlantis T3 C18 column (5 µm, 4.6 mm ╳ 150 mm). All unknown compounds in the reaction mixtures were confirmed by spiking experiments with authentic compounds either purchased from Sigma-Aldrich or synthesized in house using conventional synthetic chemistry. ^{1}H and ^{13}C NMR spectra were acquired using a Bruker Ultrashield 400 Plus operating at 400.1 MHz and 100.6 MHz respectively. Samples consisting of H_2O/D_2O mixtures were analysed using HOD suppression to collect ^{1}H NMR data. Chemical shifts (δ) are shown in ppm. The conversion yields were determined by relative integrations of the signals in the ^{1}H NMR spectrum. Coupling constants (J) are given in Hertz (Hz) and the notations s, d, m represent the multiplicities singlet, doublet, and multiplet signal. Mass spectra were recorded with an Agilent Technologies 6130 Quadrupole LC-MS using positive and negative Electron Spray Ionisation. *UV-Vis* spectra were recorded using a NanoDrop ND-1000 spectrophotometer.

Synthetic Procedures

Photoreaction of thioanhydroadenosine 6 with bisulfite/sulfite at different pH values.

Thioanhydroadenosine **6** (2 mg, 0.007 mmol) was mixed with Na_2SO_3 (4 mg, 0.032 mmol) in 0.5 mL of H_2O (containing 10% D_2O) and the pH of mixture was adjusted to the designated pH (pH = 8, 9 or 10) with 1M HCl or 1M NaOH. The mixture was then transferred to a Suprasil quartz NMR tube and irradiated at 254 nm for 2-20 hours. The mixture was periodically removed from the reactor to record ^{1}H NMR spectra. Yields were determined based on relative integrations of the signals referred to an internal standard (4 or 5 ul of 25mM pentaerythritol in H_2O) in ^{1}H NMR spectra. Yields for the various products are listed in Table 1.

Preparation and characterization of 8-mercaptoadenosine-2'-α-sulfonate 11 and 8-mercaptoadenosine-2'-β-sulfonate 12.

Thioanhydroadenosine **6** (20 mg, 0.070 mmol) was mixed with Na_2SO_3 (40 mg, 0.32 mmol) in 5 ml of H_2O (containing 10% D_2O) and the pH of mixture was adjusted to 9 with 1M NaOH. The mixture was transferred to a Suprasil quartz tube (purchased from Cambridge Glassblowing Ltd) and irradiated at 254 nm for 5 hours. The crude material obtained after reaction was subjected to preparative reverse phase HPLC, using acetonitrile in water as the eluent, to afford 3.6 mg (0.010 mmol, 14.7 % yield) of 8-mercaptoadenosine-2'-α-sulfonate **14** together with 3.3 mg (0.009 mmol, 13.2% yield) of 8-mercaptoadenosine-2'-β-sulfonate **15**.

8-mercaptoadenosine-2'-α-sulfonate **14**: ^{1}H NMR (400 MHz, D2O): δ 8.11 (1H, s, H2), 7.03 (1H, d, J = 8.2 Hz, H1'), 4.90-4.78 (2H, m, H2' and H3'), 4.23 (1H, q, J = 3.2 Hz, H4'), 3.96-3.75 (2H, m, H5'); ^{13}C NMR (101 MHz, D_2O) δ 167.6 (C4), 152.1 (C2), 148.3 (C6), 148.2 (C8), 108.1 (C5), 86.9 (C4'), 85.4 (C1'), 71.6 (C3'), 61.9 (C5'), 61.1 (C2'); ESI-LCMS (neg. m/z): 362.0 $[M-H]^-$.

8-mercaptoadenosine-2'-β-sulfonate **15**: ^{1}H NMR (400 MHz, D_2O) δ 8.08 (1H, s, H2), 7.12 (1H, d, J = 8.5 Hz, H1'), 5.14 (1H, dd, J = 9.9, 8.2 Hz, H3'), 4.26 – 3.69 (4H, m, H2', H4' and H5'); ^{13}C NMR (101 MHz, D_2O) δ 167.3 (C4), 151.3 (C2), 149.1 (C8), 148.1 (C6), 107.9 (C5), 83.8 (C4'), 83.4 (C1'), 69.7 (C3'), 66.3 (C2'), 59.9 (C5'); ESI-LCMS (neg. m/z): 362.0 $[M-H]^-$.

Preparation and characterization of adenosine-2'-β-sulfonate 13.

8-Mercaptoadenosine-2'-β-sulfonate **15** (4.0 mg, 0.011 mmol) was dissolved in 1 mL of degassed H_2O (containing 10% D_2O). The mixture was transferred to a Suprasil quartz NMR tube and irradiated at 254 nm for 24 hours, after which all starting material was consumed. The resulting suspension was filtered and the filtrate lyophilized. The residue was dissolved in 0.5 mL of D_2O without further purification and the adenosine-2'-β-sulfonate **15** (3.5 mg, 95% yield) obtained was characterized by 1D and 2D NMR spectroscopy: ^{1}H NMR (400 MHz, D_2O) δ 8.22 (1H, s, H8), 8.17 (1H, s, H2), 6.61 (1H, d, J = 7.8 Hz, H1'), 4.79 (1H, t, J = 8.6 Hz, H3'), 4.06 – 3.88 (4H, m, H2', H4', H5'); ^{13}C NMR (101 MHz, D_2O) δ 154.8 (C6), 151.3 (C2), 148.9 (C4), 142.2 (C8), 117.9 (C5), 83.7 (C4'), 82.1 (C1'), 70.1 (C3'), 66.4 (C2'), 59.6 (C5'); ESI-LCMS (neg. m/z): 330.0 $[M-H]^-$.

Preparation and characterization of adenosine-2'-α-sulfonate 12.

8-Mercaptoadenosine-2'-α-sulfonate **14** (5.0 mg, 0.014 mmol) was dissolved in 1 mL of degassed H_2O (containing 10% D_2O). The mixture was transferred to a Suprasil quartz NMR tube and irradiated at 254 nm for 24 hours, after which all starting material was consumed. The resulting suspension was filtered and the filtrate lyophilized. The residue was dissolved in 0.5 mL of D_2O without further purification and the adenosine-2'-α-sulfonate **12** (4.5 mg, 97% yield) obtained was characterized by 1D and 2D NMR spectroscopy: ^1H NMR (400 MHz, D_2O) δ 8.29 (1H, s, H8), 8.20 (1H, s, H2), 6.54 (1H, d, J = 8.9 Hz, H1'), 4.76 (1H, dd, J_1 = 5.8 Hz, J_2 = 2.2 Hz, H3'), 4.37 (1H, dd, J_1 = 8.9 Hz, J_2 = 5.8 Hz, H2'), 4.27 (1H, m, H4'), 3.82 (2H, m, H5'); ^{13}C NMR (101 MHz, D_2O) δ 155.2 (C6), 151.9 (C2), 148.4 (C4), 141.4 (C8), 119.3 (C5), 87.6 (C4'), 85.7 (C1'), 71.9 (C3'), 64.2 (C2'), 61.7 (C5'); ESI-LCMS (neg. m/z): 330.0 $[M-H]^-$.

Photoreaction of 8-mercaptoadenosine 11.

8-Mercaptoadenosine **11** (5.0 mg, 0.017 mmol) was dissolved in 1 mL of H_2O (containing 10% D_2O). The mixture was transferred to a Suprasil quartz NMR tube and irradiated at 254 nm for 12 hours, after which all starting material was consumed. The resulting suspension was filtered and the filtrate lyophilized. 4.2 mg (95% yield) of adenosine **8** was obtained.

Photoreaction of thioanhydroadenosine 6 at pH 9.

Thioanhydroadenosine **6** (2.0 mg, 0.007 mmol) was dissolved in 0.5 mL of H_2O (containing 10% D_2O) and the pH of the mixture was adjusted to 9 with 1M NaOH. The mixture was transferred to a Suprasil quartz NMR tube and irradiated at 254 nm for 5 hours. The sample was removed from the reactor to record the ^1H NMR spectrum at the end of reaction. The conversion yields were determined by relative integrations of the signals referred to an internal standard (10 uL of 25 mM pentaerythritol) in the ^1H NMR spectrum and the identity of the major products (ribal **16** [1] in 11% yield and 8-mercaptoadenine **17** in 10% yield) was confirmed by spiking experiment with an authentic standard made in house.

Irradiation of 9-β-D-arabinofuranosyl adenine 22 in the presence or absence of sulfite at pH 9.

9-β-D-Arabinofuranosyl adenine **22** (2.0 mg, 0.007 mmol) was mixed with Na_2SO_3 (4.0 mg, 0.032 mmol) in 0.5 mL of H_2O (containing 10% D_2O) and the pH of mixture was adjusted to 9 with 1M NaOH. The mixture was then transferred to a Suprasil quartz NMR tube and irradiated at 254 nm for 2 hours. The starting material was unchanged. Similarly, at pH 9, 9-β-D-Arabinofuranosyl adenosine **22** was stable to irradiation at 254 nm in the absence of Na_2SO_3.

Nitrosation of adenosine 8 with nitrous acid at pH 4.

Adenosine **8** (2.7 mg, 0.01 mmol) and NaH_2PO_4 (12.0 mg, 0.1 mmol) were dissolved in 0.9 mL of H_2O (containing 10% D_2O) and the pH of mixture was adjusted to 4 with 1M HCl. Nitrous acid (0.1 mL of a 1M solution of nitrous acid at pH 4 freshly prepared from $NaNO_2$ acidified with 1M HCl) in H_2O (containing 10% D_2O) was then added and the mixture was stirred at room temperature for the duration of the experiment. A set amount of pentaerythritol as internal standard was added to the mixture at the start of reaction. Yields are based on the relative integration referred to the internal standard. Aliquots were removed periodically for analysis by

^1H NMR spectroscopy. The starting material was converted to a 51:49 mixture of adenosine **8** and inosine **9** (47% and 46% yield, respectively).

Nitrosation of 8-mercaptoadenosine 11 with nitrous acid at pH 4.

8-Mercaptoadenosine **11** (2.8 mg, 0.01 mmol) and NaH_2PO_4 (12.0 mg, 0.1 mmol) were dissolved in 0.9 mL of H_2O (containing 10% D_2O) and the pH of mixture was adjusted to 4 with 1M HCl. Nitrous acid (0.1 mL of a 1M solution of nitrous acid at pH 4 freshly prepared from $NaNO_2$ acidified with 1M HCl) in H_2O (containing 10% D_2O) was then added and the mixture was stirred at room temperature for the duration of the experiment. A set amount of pentaerythritol as internal standard was added to the mixture at the start of reaction. Yields are based on the relative integration referred to the internal standard. Aliquots were removed periodically for analysis by ^1H NMR spectroscopy. The starting material was converted to a mixture of adenosine **8**, inosine **9**, and 5', 8-cycloadenosine **24**[2], which decomposed after 12 days, leaving a mixture of **8** and **9** in a ratio of 76:24 (71% and 23% yield, respectively). [Note that the reaction becomes temporarily gelatinous following the addition of nitrous acid]

Nitrosation of a 1:3 mixture of 8-mercaptoadenosine 11 and 8-mercapto-2'-deoxyadenosine 10 with nitrous acid and hydrolysis of the products in phosphate buffer at pH 4.

8-Mercaptoadenosine **11** (0.7 mg, 0.0025 mmol), 8-mercapto-2'-deoxyadenosine **10** (2.0 mg, 0.075 mmol) and NaH_2PO_4 (12.0 mg, 0.1 mmol) were dissolved in 0.9 mL of H_2O (containing 10% D_2O) and the pH of mixture was adjusted to 4 with 1M HCl. Nitrous acid (0.1 mL of a 1M solution of nitrous acid at pH 4 freshly prepared from $NaNO_2$ acidified with 1M HCl) in H_2O (containing 10% D_2O) was then added and the mixture was stirred at room temperature for the duration of the experiment. In contrast to the previous experiments, the pH was readjusted to 4 every 24 hours. A set amount of pentaerythritol as internal standard was added to the mixture at the start of reaction. Yields are based on the relative integration referred to the internal standard. Aliquots were removed periodically for analysis by ^1H NMR spectroscopy. The starting material was converted to a mixture of adenosine **8**, inosine **9**, 2'-deoxyadenosine **1**, and 2'-deoxyinosine **2** in a ratio of 17:7:49:27 after 12 days (17%, 6%, 48%, and 27% yield, respectively). [Note that the reaction becomes temporarily gelatinous following the addition of nitrous acid]

Photoreaction of 2'-deoxy-β-bromoadenosine 23 at pH 9 with sulfite.

2'-deoxy-β-bromoadenosine **23** (2 mg, 0.006 mmol) was mixed with Na_2SO_3 (4 mg, 0.032 mmol) in 0.5 mL of H_2O (containing 10% D_2O) and the pH of mixture was adjusted to 9 with 1M NaOH. The mixture was then transferred to a Suprasil quartz NMR tube and irradiated at 254 nm for 3 hours. The mixture was periodically removed from the reactor to record ^1H NMR spectra. The identities of products were confirmed by spiking experiments with authentic standards in ^1H NMR spectra. Yields were determined based on relative integrations of the signals referred to an internal standard (25 ul of 25mM pentaerythritol in H_2O) in ^1H NMR spectra.

Control experiment with 2'-deoxy-β-bromoadenosine 23 and sulfite at pH 9 in the dark.

2'-deoxy-β-bromoadenosine **23** (2 mg, 0.006 mmol) was mixed with Na_2SO_3 (4 mg, 0.032 mmol) in 0.5 mL of H_2O (containing 10% D_2O) and the pH of mixture

was adjusted to 9 with 1M NaOH. The mixture was monitored by ^1H NMR spectra. No adenosine **8**, arabino-adenosine **22** or sulfonates **12**/**13** were detected in the mixture after 3 hours in the dark.

Reactions with thioanhydroadenosine **6**

Positive labelling experiment (Rxn 2)

Thioanhydroadenosine **6** (2 mg, 7.1 μmol) was dissolved in 0.45 mL H_2O (containing 10% D_2O) and adjusted to pH 11. A concentrated solution of ^{18}O-labelled sulfite was prepared by dissolving 36 mg $NaHSO_3$ in 300 μL $H_2{}^{18}O$ (initial pH 5, exchange fast) and allowing to sit for 1h. This solution was then adjusted to pH 13 by adding solid potassium hydroxide. 28 μL of the sulfite solution (0.032 mmol) was then added to the solution of **6**. The pH was adjusted from 12.5 to 11.2 and the sample was immediately transferred to a quartz NMR tube and irradiated at 254 nm. An aliquot was taken at 25 min for LCMS, and after 65 min, 1H-NMR and LCMS were recorded. LCMS and standards were used to verify the identity of product peaks. ^{18}O-Labelling of sulfonates **14** and **15** and 8-mercaptoadenosine **11** was observed.

Control Experiment (Rxn 1)

An identical experiment to Rxn 2 was performed using unlabelled water in the preparation of the sulfite solution.

Negative labelling experiment (Rxn 3)

Thioanhydroadenosine **6** (2 mg, 7.1 μmol) was dissolved in 0.45 mL $H_2{}^{18}O$ and adjusted to pH 12 using 5M NaOH. A concentrated solution of unlabelled sulfite was prepared by dissolving 89 mg Na_2SO_3 in H_2O, and adjusting the pH to 11. Then, 45 μL of the sulfite solution (0.032 mmol) was then added to the solution of **6**. The pH was adjusted down to 11.2 and the sample was immediately transferred to a quartz NMR tube and irradiated at 254 nm. An aliquot was taken at 25 min for LCMS, and after 65 min, 1H-NMR and LCMS were recorded. Sulfonates **14** and **15** and 8-mercaptoadenosine **11** were observed without ^{18}O-labelling.

Reactions with 2'-deoxy-2'-β-bromoadenosine **23**

Control Experiment (Rxn 4)

An identical experiment to Rxn 1 was performed, but replacing thioanhydroadenosine **6** with 2'-deoxy-2'-β-bromoadenosine **23** (1 mg, 3.0 μmol). *Ribo*-adenosine **8**, *arabino*-adenosine **22**, and α- and β-sulfonates (**12, 13**) were detected by LCMS, verified by standards.

Positive labelling experiment (Rxn 5)

An identical experiment to Rxn 2 was performed, but replacing thioanhydroadenosine **6** with 2'-deoxy-2'-β-bromoadenosine **23** (1 mg, 3.0 μmol). O^{18}-Labelling of sulfonates **12** and **13** and adenosines **8** and **22** was observed.

Negative labelling experiment (Rxn 6)

An identical experiment to Rxn 3 was performed, but replacing thioanhydroadenosine **6** with 2'-deoxy-2'-β-bromoadenosine **23** (1 mg, 3.0 μmol). Sulfonates **12** and **13** and adenosines **8** and **22** was observed without ^{18}O-labelling.

Betts and Voss calculated half-times for exchange of 25.3 h and 1.3 min for oxygen exchange between aqueous sulfite and water at pH 10.5 and pH 8.9, respectively.[3]

O^{18} Labelling Experiments

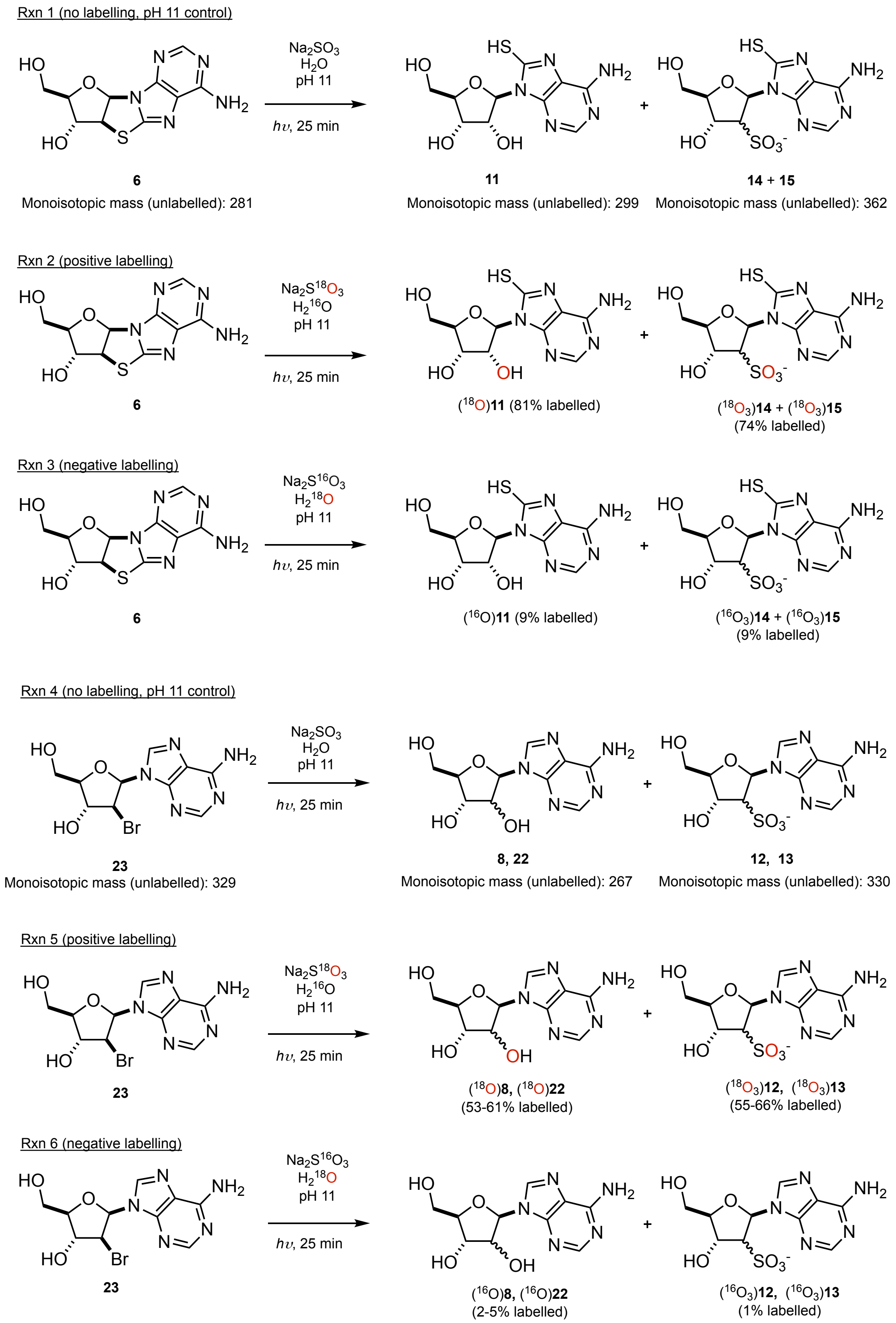

Scheme S1, labelling results from mechanism investigation.

Labelling outcomes

8-mercaptoadenosine 11, Negative ESI, Table S1

	Mass and relative abundance							
	298	299	300	301	302	Avg. mass	Δ Avg. mass	% Labelled
**Rxn 1**	84	11	5	-	-	298.2		
**Rxn 2**	28	4	58	7	3	299.8	1.62	**81**
**Rxn 3**	77	11	12	2	-	298.4	0.18	**9**

8-mercapto-2'-sulfonate 14/15, Negative ESI, Table S2

	Mass and relative abundance												
	362	363	364	365	366	367	368	369	370	371	Avg. mass	Δ Avg. mass	% Labelled
**Rxn 1**	80	10	8	1							362.3		
**Rxn 2**	2	-	8	-	28	3	53	1	5	1	366.7	4.41	**74**
**Rxn 3**	63	8	19	2	5	1	1				362.9	0.55	**9**

Arabino-adenosine 22, Positive ESI, Table S3

	Mass and relative abundance							
	268	269	270	271	272	Avg. mass	Δ Avg. mass	% Labelled
**Rxn 4**	88	11	2			268.1		
**Rxn 5**	34	5	54	7	1	269.4	1.22	**61**
**Rxn 6**	87	10	3			268.2	0.031	**2**

Adenosine 8, Positive ESI, Table S4

	Mass and relative abundance							
	268	269	270	271	272	Avg. mass	Δ Avg. mass	% Labelled
**Rxn 4**	89	9	1			268.1		
**Rxn 5**	41	5	47	6	1	269.2	1.06	**53**
**Rxn 6**	84	10	6			268.2	0.097	**5**

β-Sulfonate 13, Negative ESI, Table S5

	**Mass and relative abundance**												
	**330**	**331**	**332**	**333**	**334**	**335**	**336**	**337**	**338**	**339**	**Avg. mass**	**Δ Avg. mass**	**% Labelled**
**Rxn 4**	85	10	6								330.2		
**Rxn 5**	7	1	17	2	29	4	32	4	2	0.3	333.5	3.27	55
**Rxn 6**	81	11	8								330.3	0.057	1

α-Sulfonate 12, Negative ESI, Table S6

	**Mass and relative abundance**												
	**330**	**331**	**332**	**333**	**334**	**335**	**336**	**337**	**338**	**339**	**Avg. mass**	**Δ Avg. mass**	**% Labelled**
**Rxn 4**	84	10	6								330.2		
**Rxn 5**	8	1	18	2	29	4	32	4	2	0.2	334.2	3.96	66
**Rxn 6**	82	11	7								330.3	0.046	1

Tables S1-S6. Relative abundances are derived from low-res LCMS spectra and are therefore approximate. **Avg. mass** is the weighted average of relative abundance and signal mass. **Δ Avg. mass** is the difference between average mass of the entry and of the control experiment. **% Labelled** is the Δ Avg. mass of labelled material divided by the expected mass increase for completely oxygen-labelled material. Differences between idealized labelling and observed labelling are attributable to partial isotopic exchange between water and sulfite, which, although slow at room temperature and high pH (see overleaf), may be faster under the photochemical reaction conditions.

Supplementary Figures

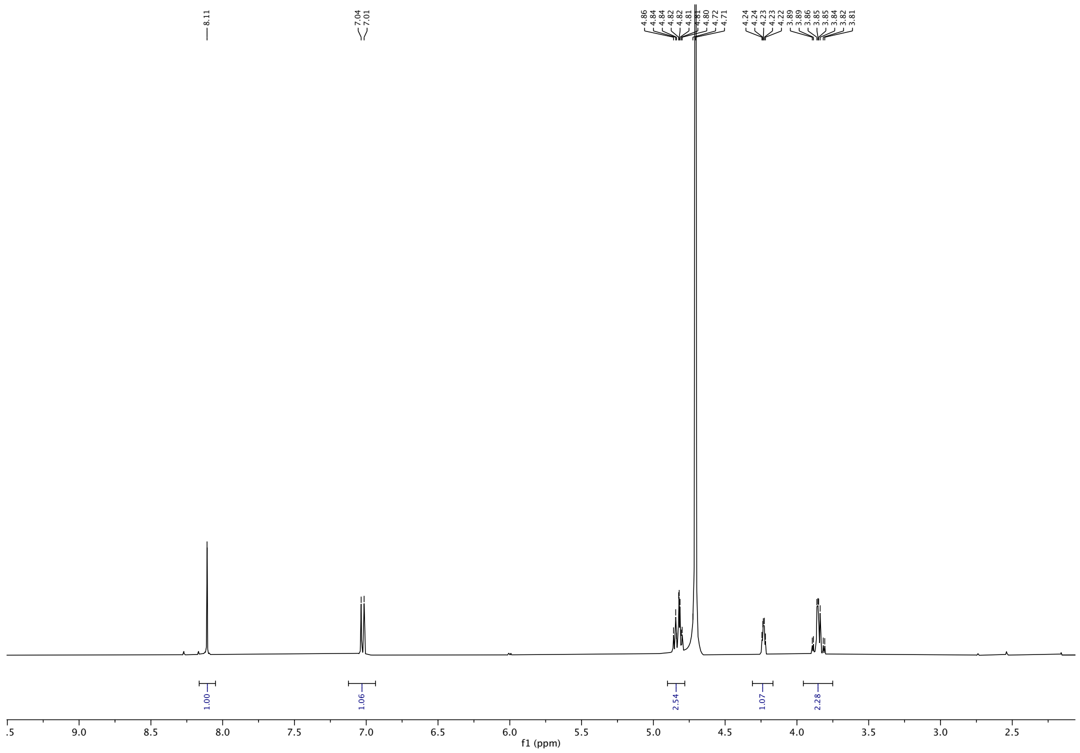

Fig. S1 ^{1}H NMR spectrum of 8-mercaptoadenosine-2'-α-sulfonate **14** in D_2O.

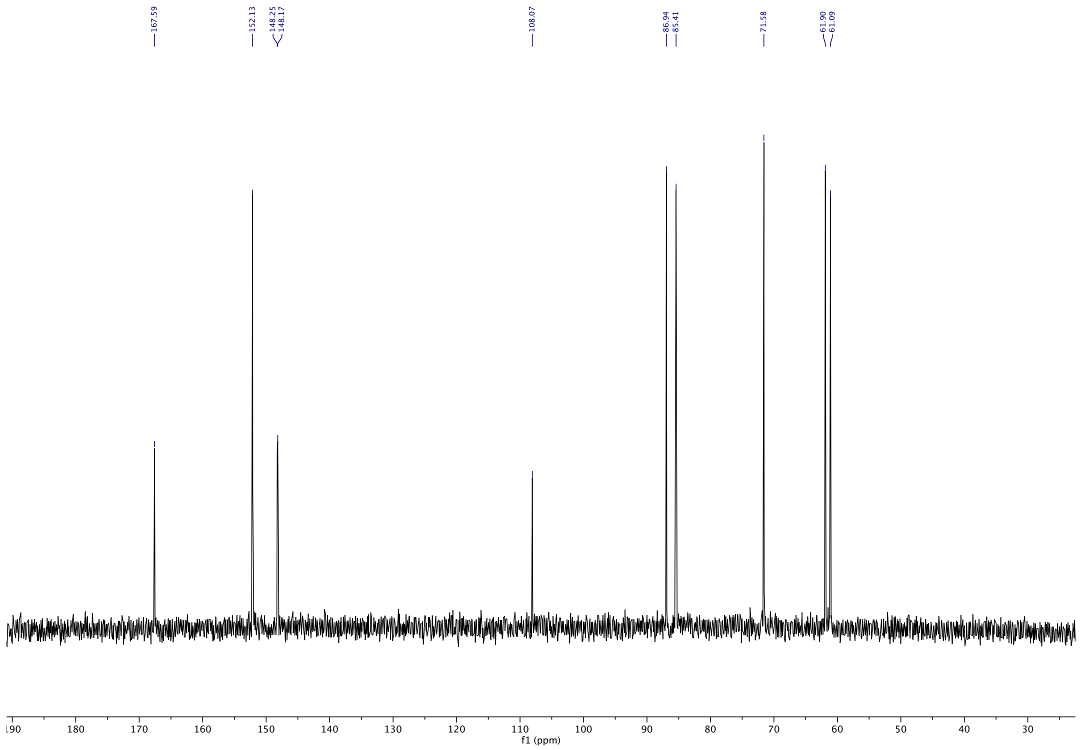

Fig. S2 ^{13}C NMR spectrum of 8-mercaptoadenosine-2'-α-sulfonate **14** in D_2O.

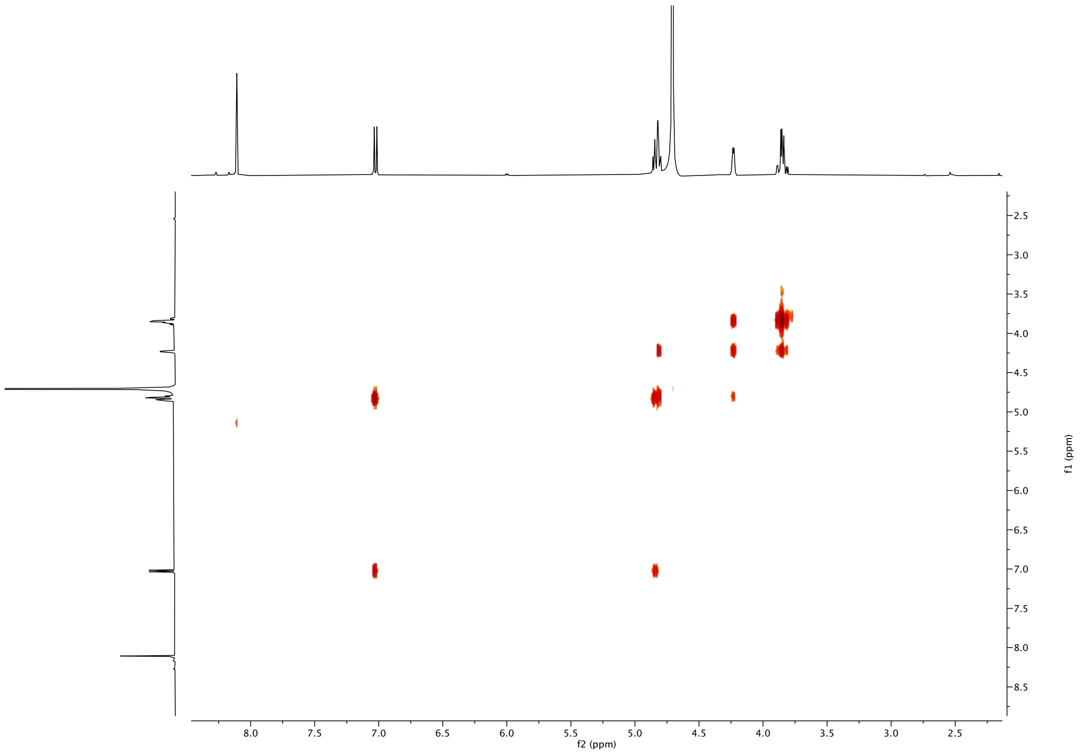

Fig. S3 COSY NMR spectrum of 8-mercaptoadenosine-2'-α-sulfonate **14** in D_2O.

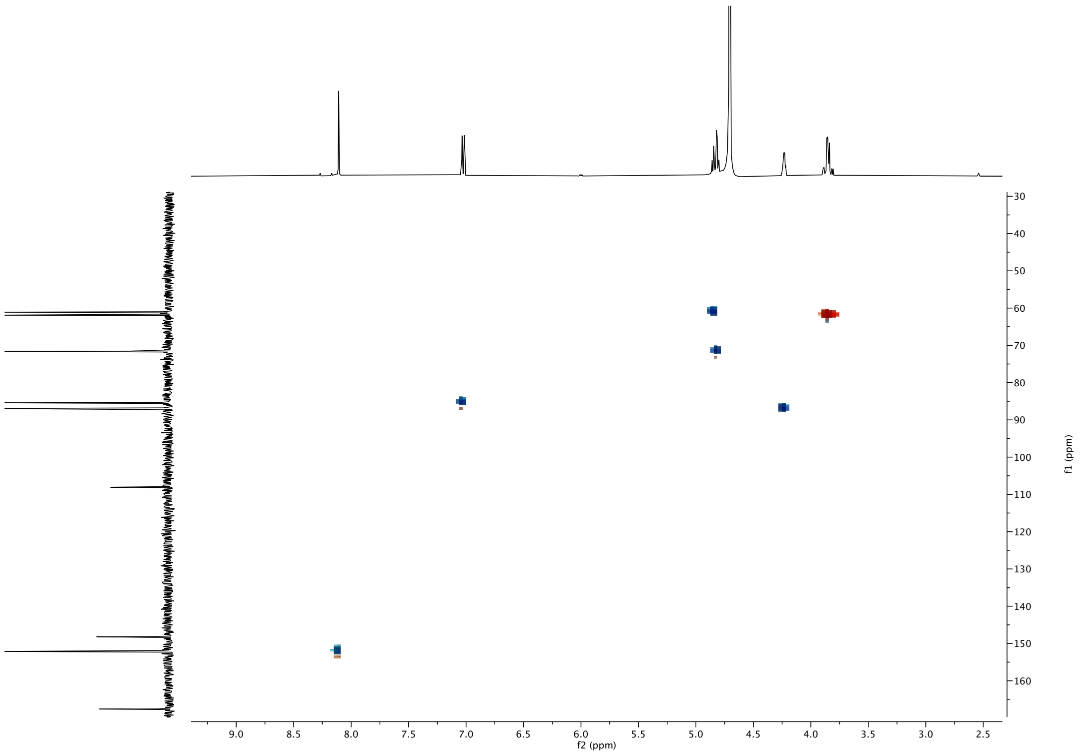

Fig. S4 HSQC NMR spectrum of 8-mercaptoadenosine-2'-α-sulfonate **14** in D_2O.

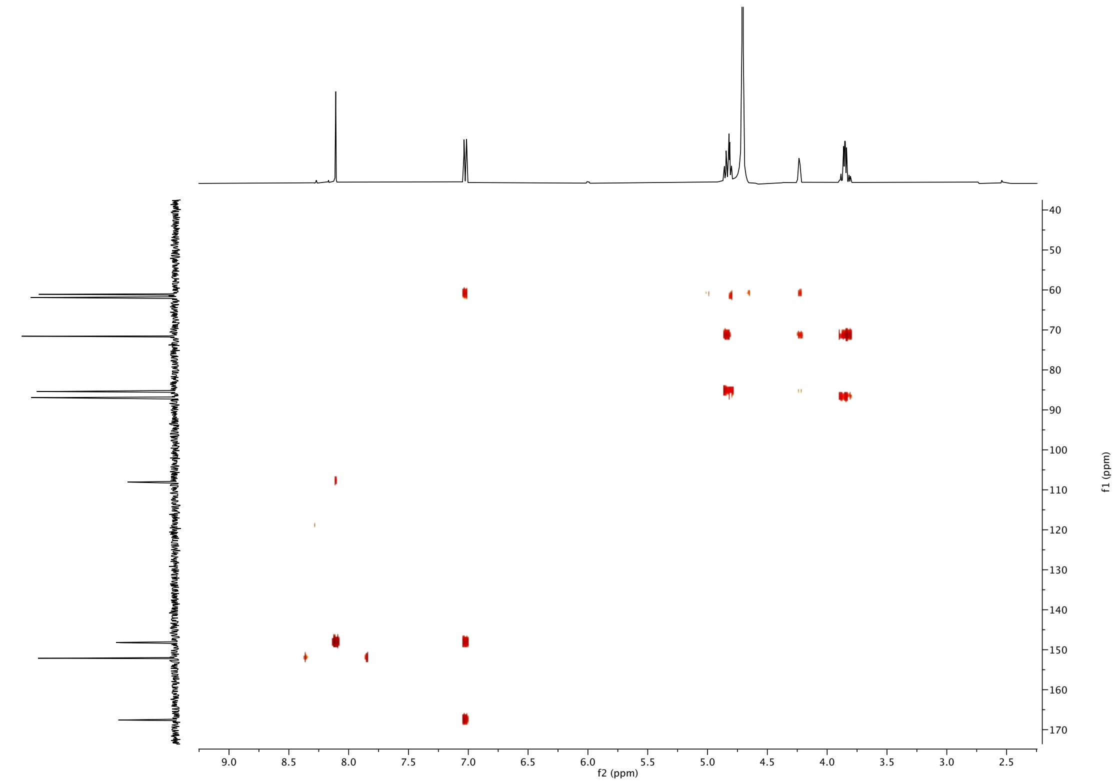

Fig. S5 HMBC NMR spectrum of 8-mercaptoadenosine-2'-α-sulfonate **14** in D_2O.

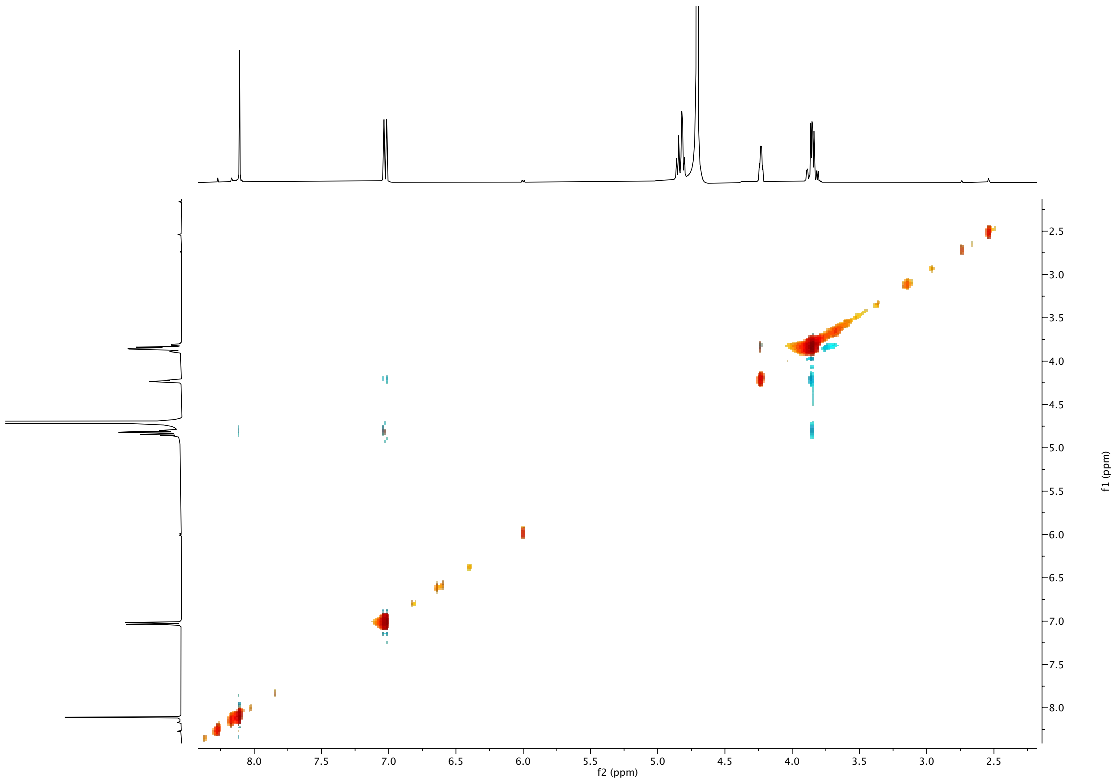

Fig. S6 NOESY NMR spectrum of 8-mercaptoadenosine-2'-α-sulfonate **14** in D_2O.

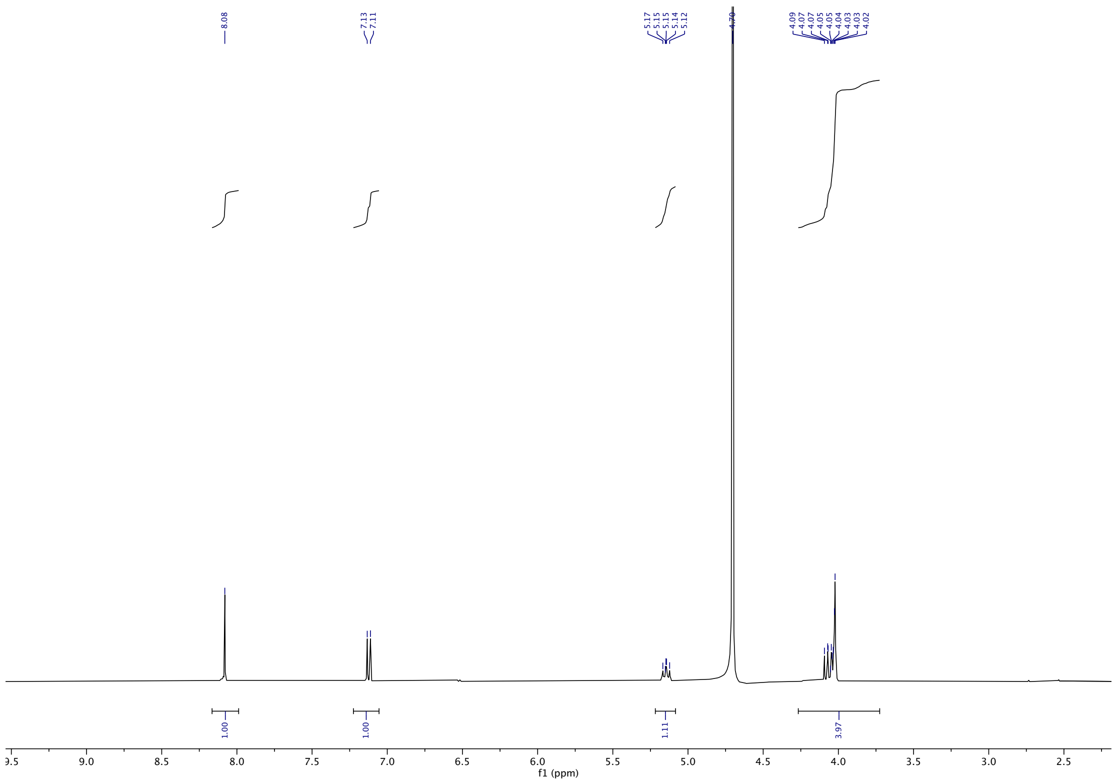

Fig. S7 ^{1}H NMR spectrum of 8-mercaptoadenosine-2'-β-sulfonate **15** in D_2O.

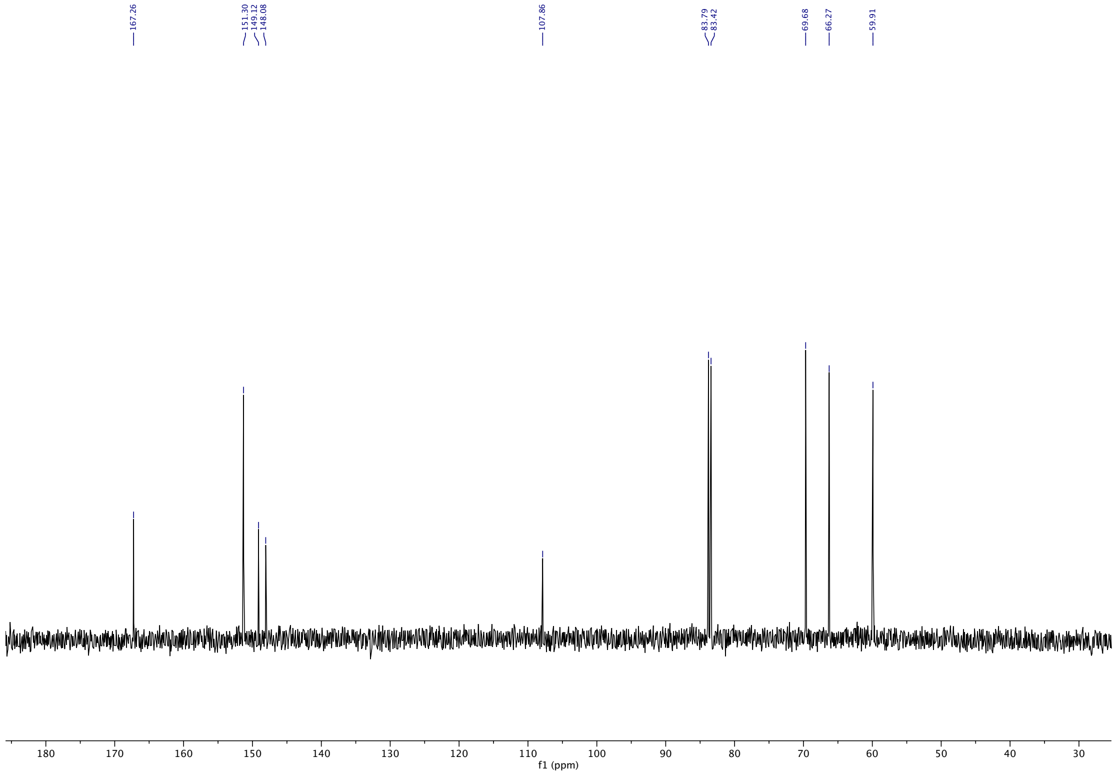

Fig. S8 ^{13}C NMR spectrum of 8-mercaptoadenosine-2'-β-sulfonate **15** in D_2O.

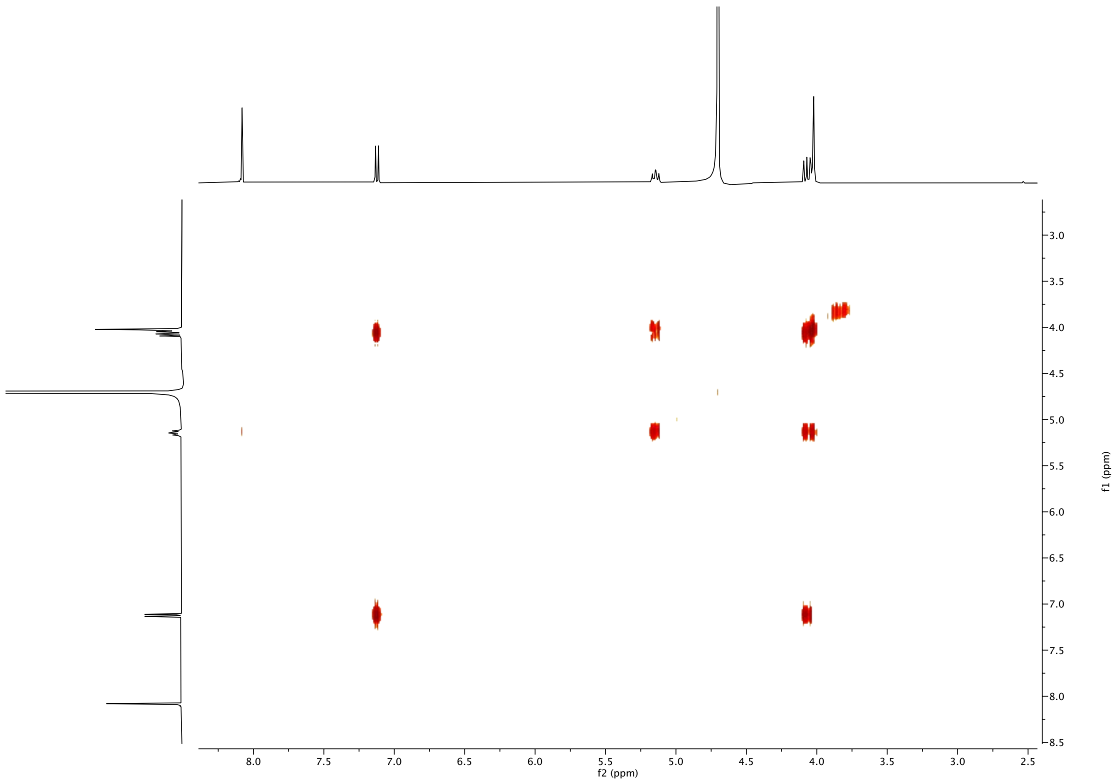

Fig. S9 COSY NMR spectrum of 8-mercaptoadenosine-2'-β-sulfonate **15** in D_2O.

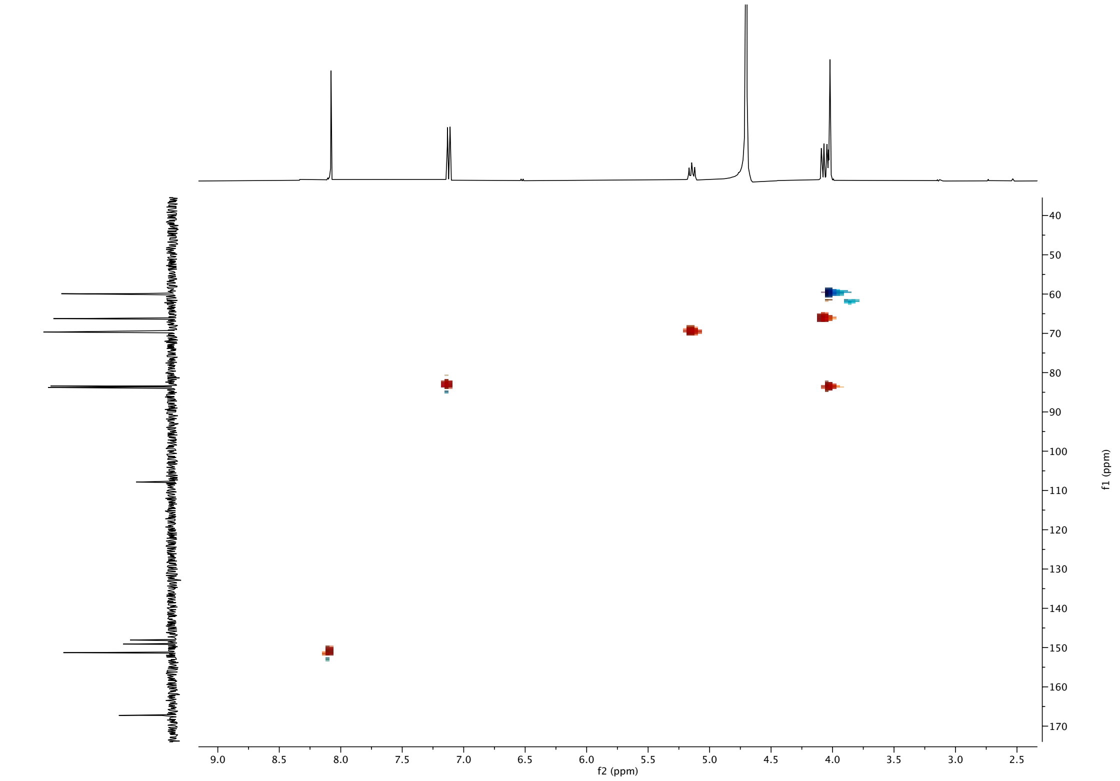

Fig. S10 HSQC NMR spectrum of 8-mercaptoadenosine-2'-β-sulfonate **15** in D_2O.

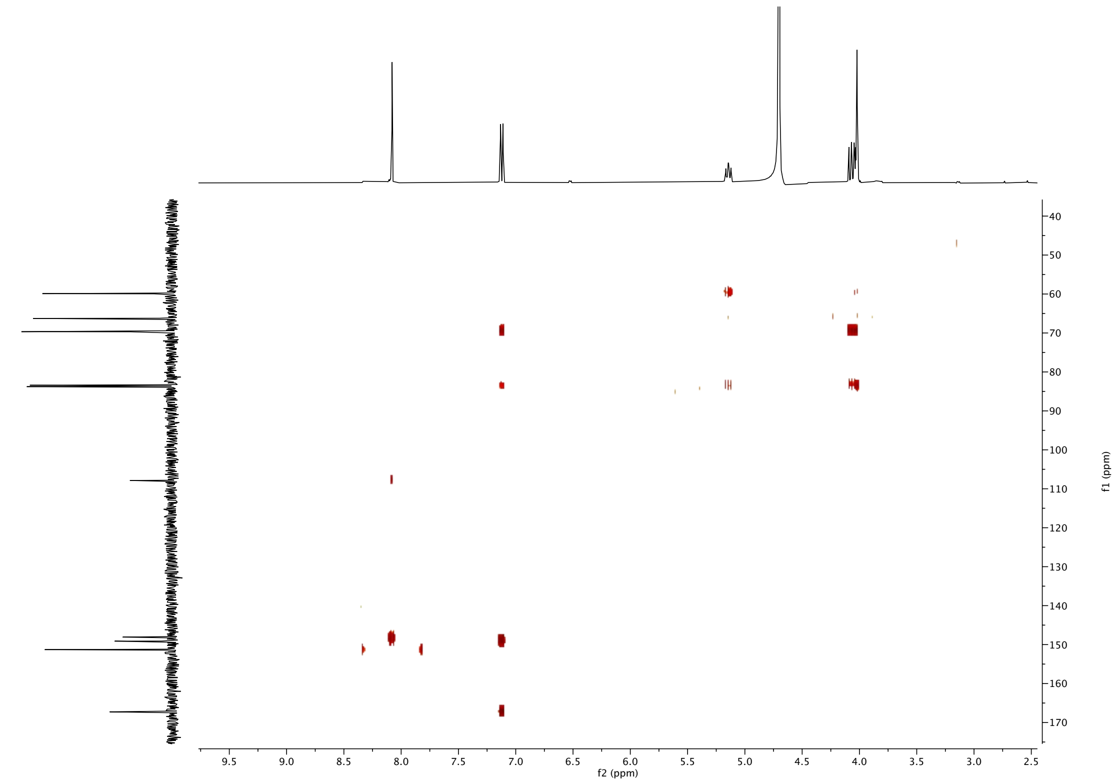

Fig. S11 HMBC NMR spectrum of 8-mercaptoadenosine-2'-β-sulfonate **15** in D_2O.

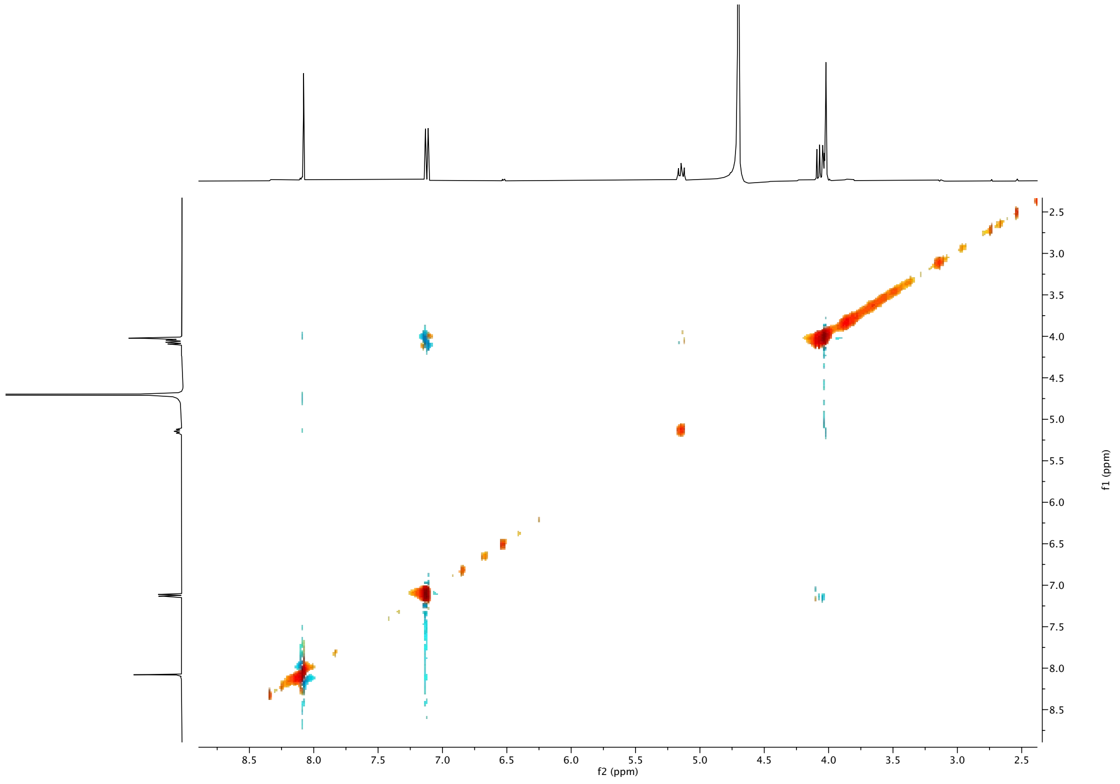

Fig. S12 NOESY NMR spectrum of 8-mercaptoadenosine-2'-β-sulfonate **15** in D_2O.

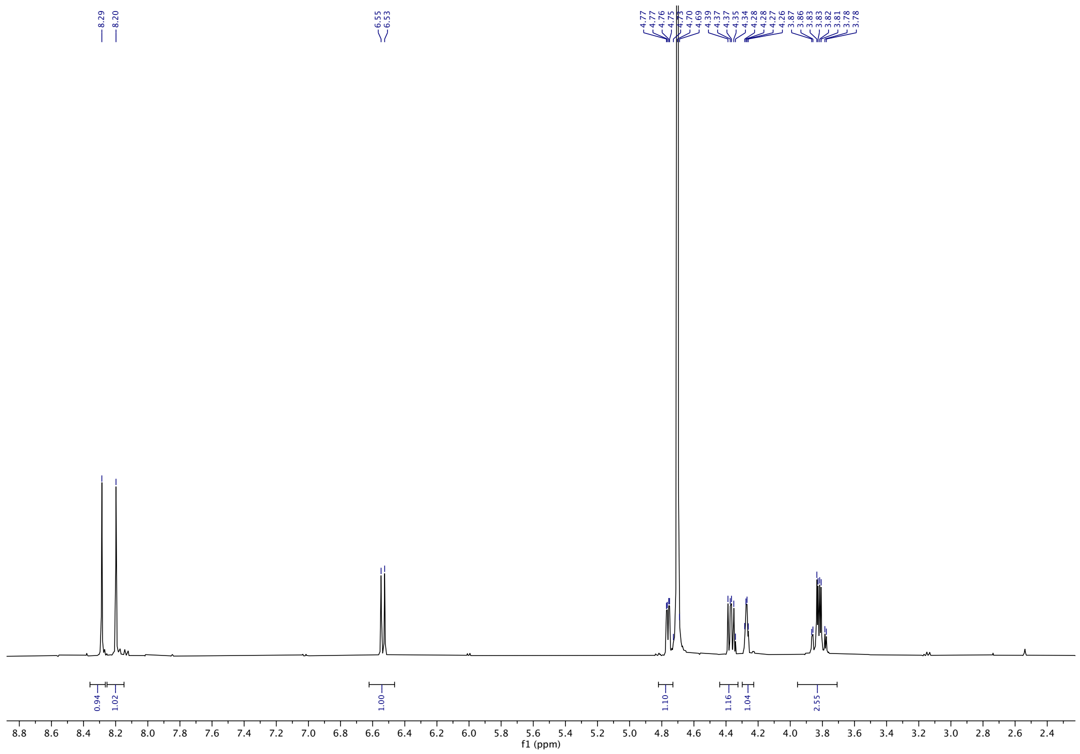

Fig. S13 ^{1}H NMR spectrum of adenosine-2'-α-sulfonate **12** in D_2O.

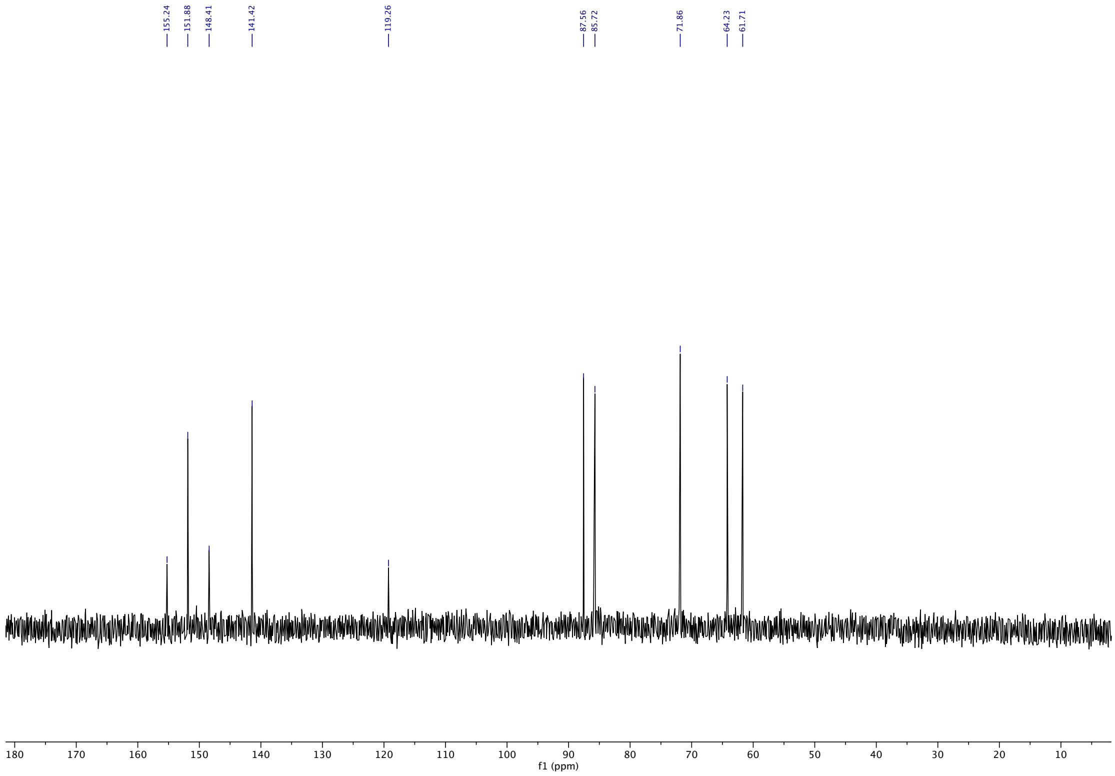

Fig. S14 ^{13}C NMR spectrum of adenosine-2'-α-sulfonate **12** in D_2O.

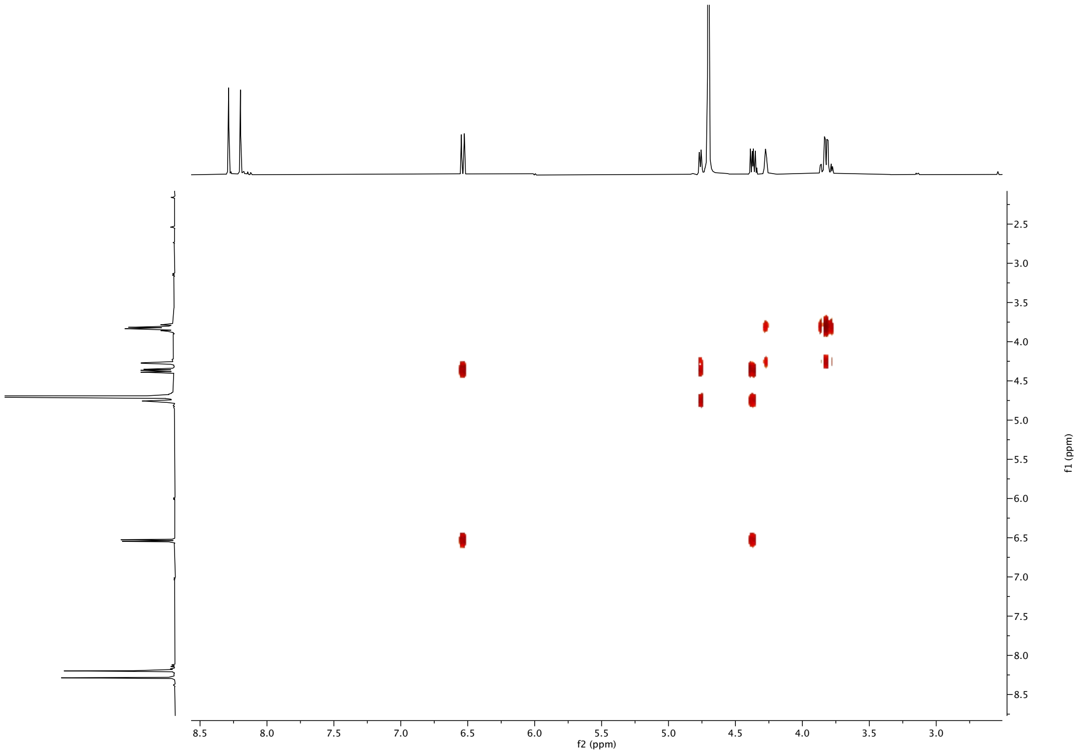

Fig. S15 COSY NMR spectrum of adenosine-2'-α-sulfonate **12** in D_2O.

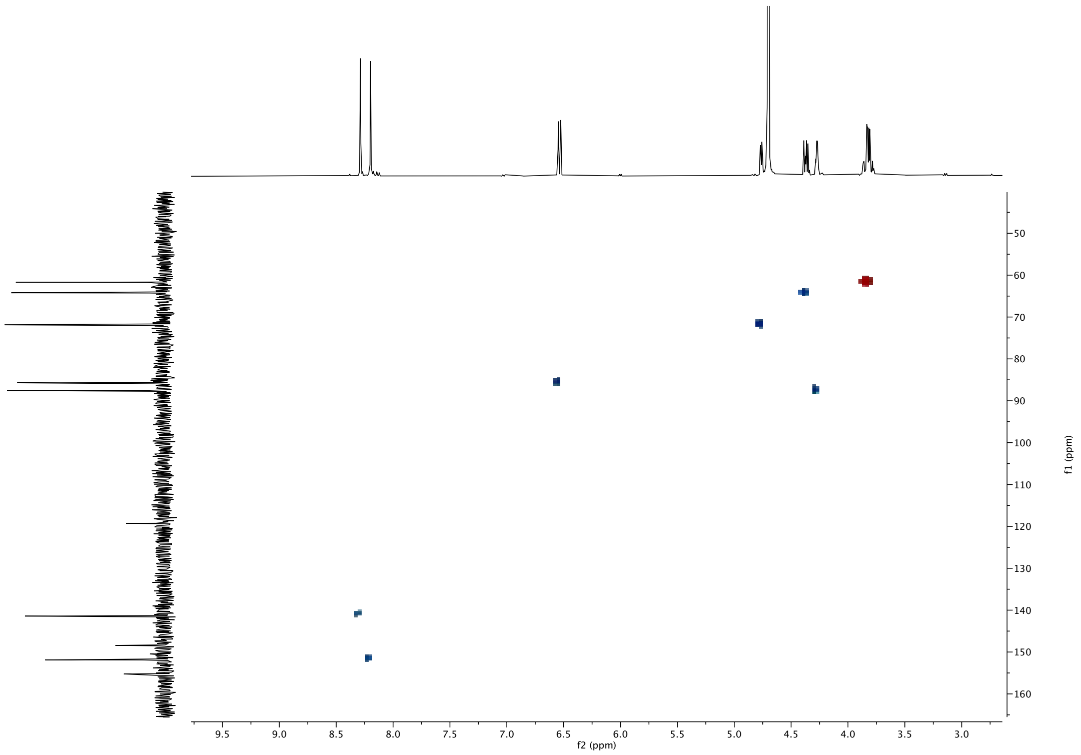

Fig. S16 HSQC NMR spectrum of adenosine-2'-α-sulfonate **12** in D_2O.

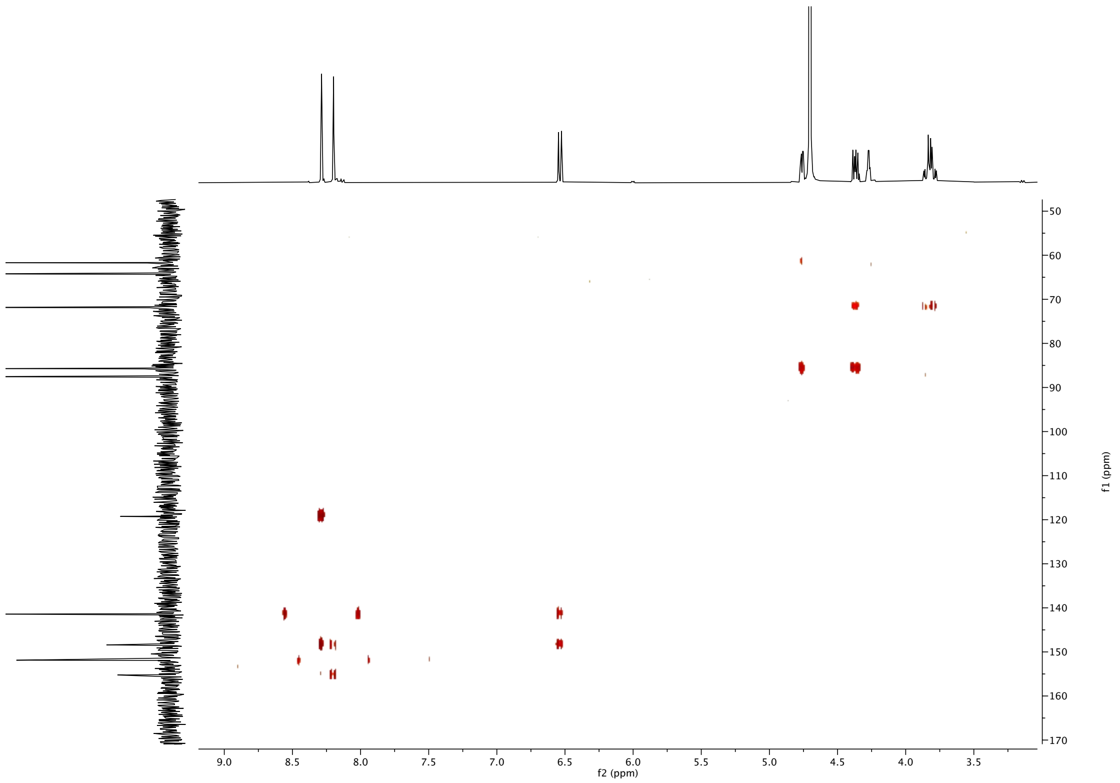

Fig. S17 HSQC NMR spectrum of adenosine-2'-α-sulfonate **12** in D_2O.

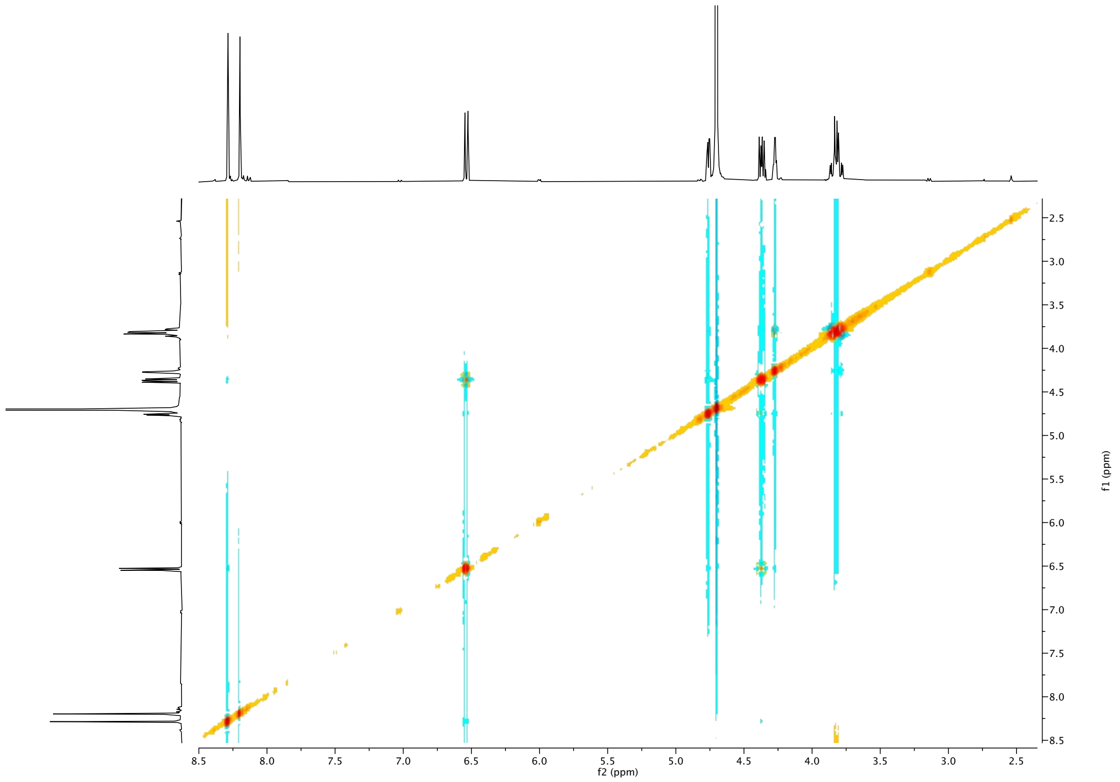

Fig. S18 NOESY NMR spectrum of adenosine-2'-α-sulfonate **12** in D_2O.

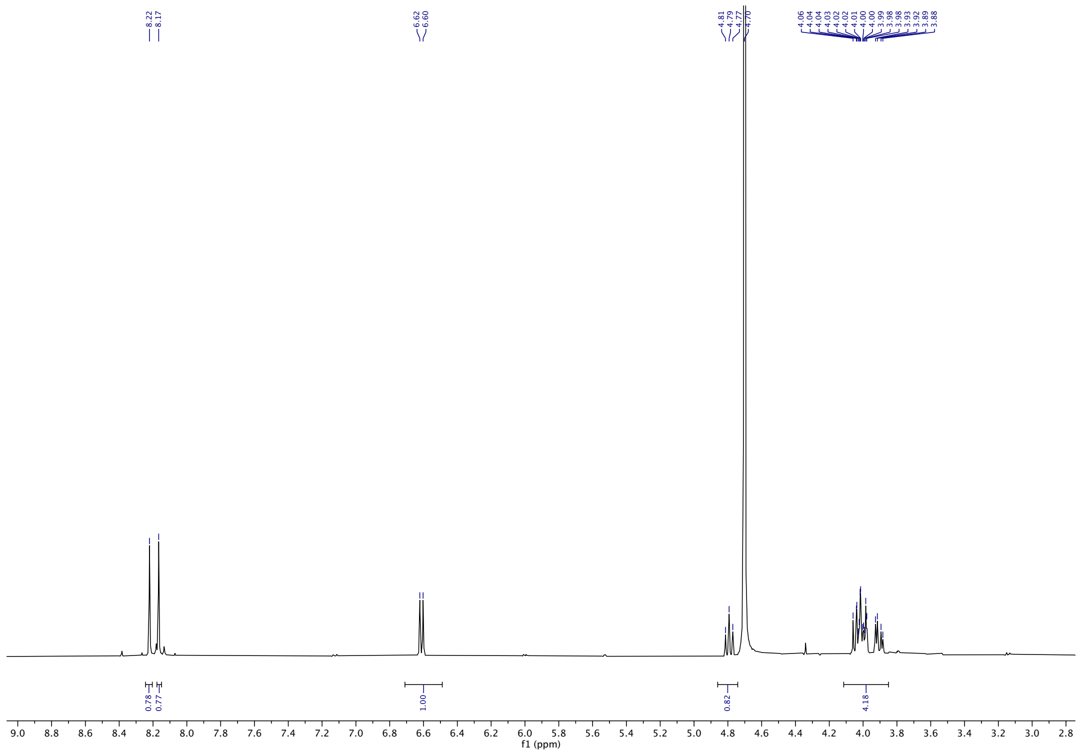

Fig. S19 ^{1}H NMR spectrum of adenosine-2'-β-sulfonate **13** in D_2O.

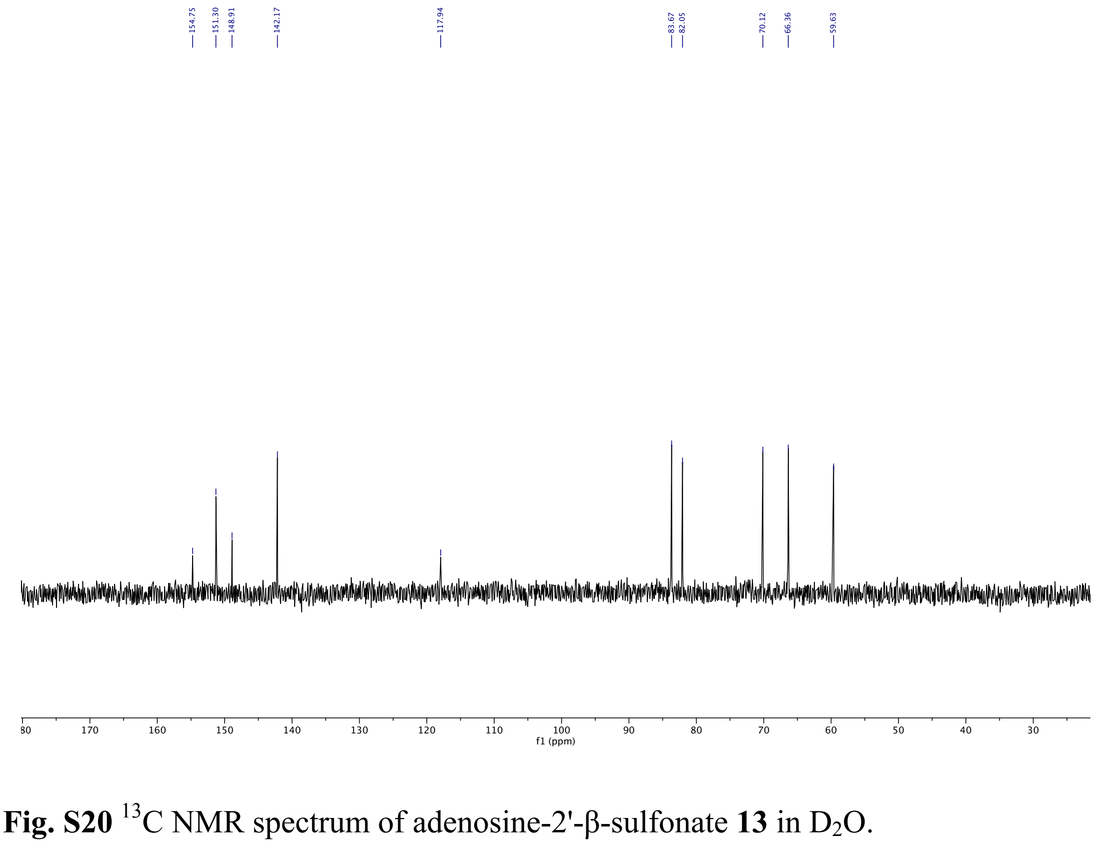

Fig. S20 ^{13}C NMR spectrum of adenosine-2'-β-sulfonate **13** in D_2O.

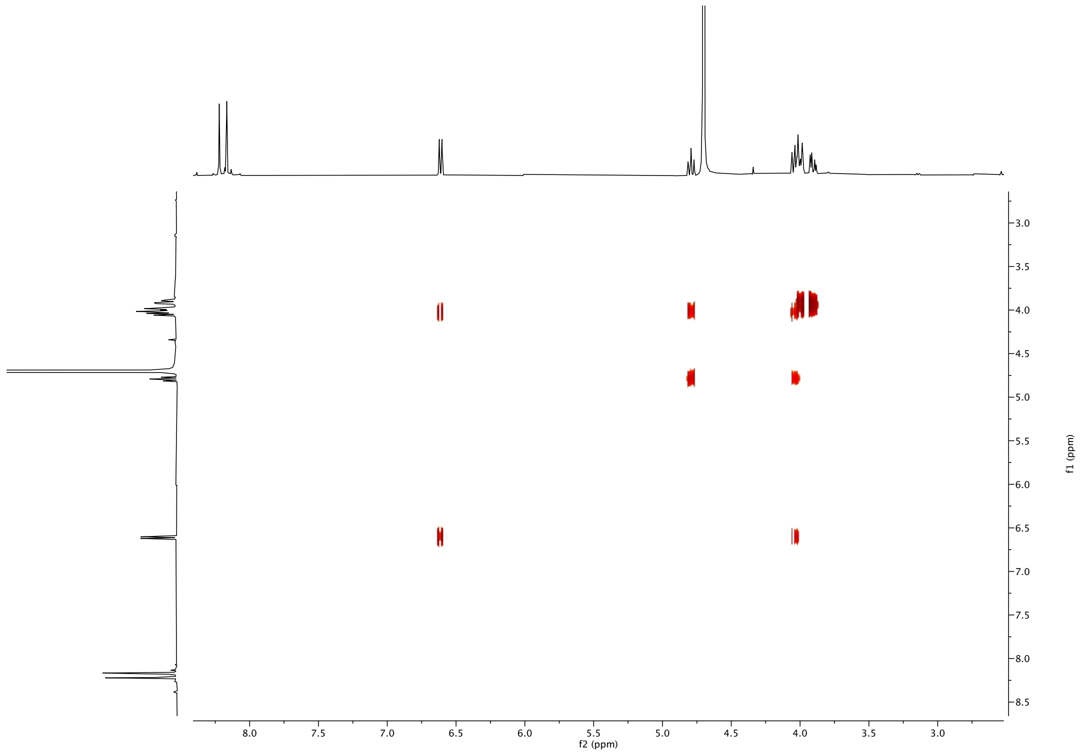

Fig. S21 COSY NMR spectrum of adenosine-2'-β-sulfonate **13** in D_2O.

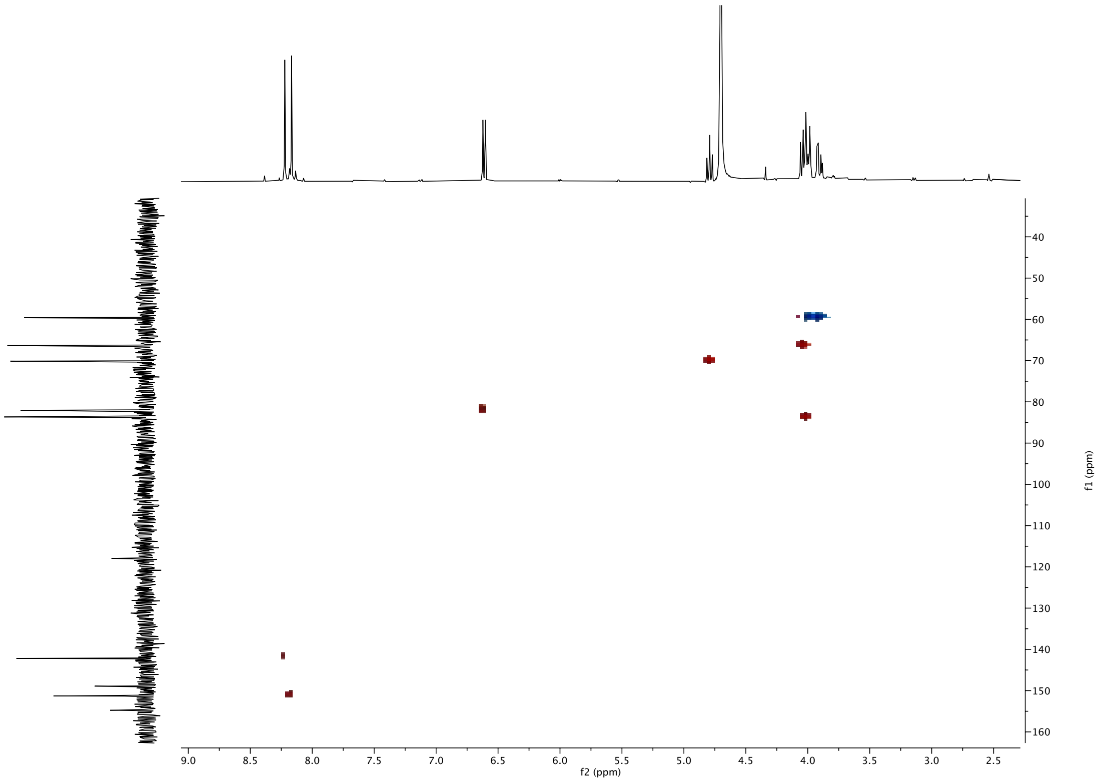

Fig. S22 HSQC NMR spectrum of adenosine-2'-β-sulfonate **13** in D_2O.

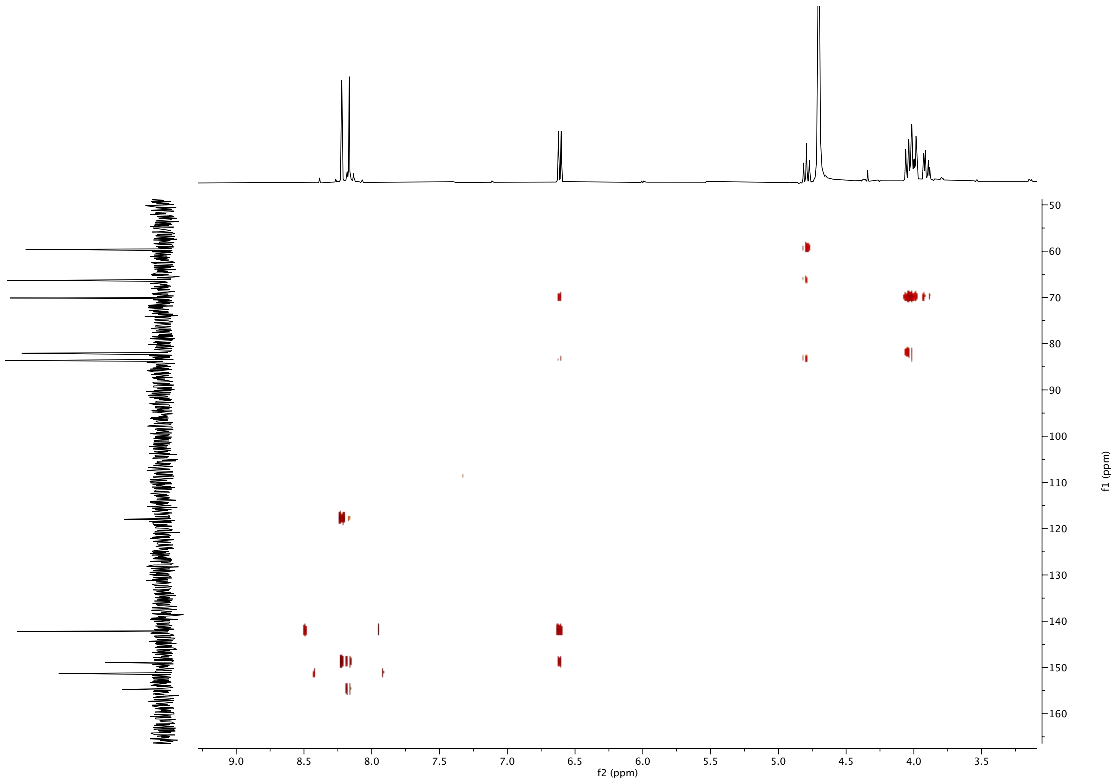

Fig. S23 HMBC NMR spectrum of adenosine-2'-β-sulfonate **13** in D_2O.

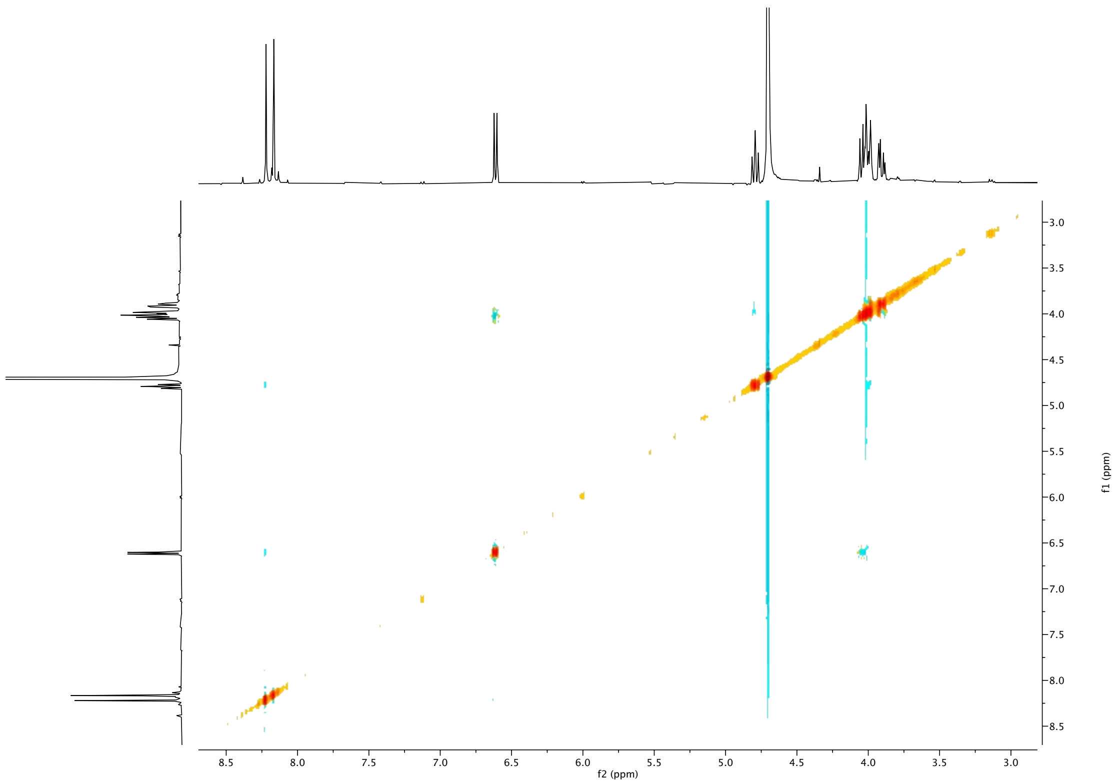

Fig. S24 NOESY NMR spectrum of adenosine-2'-β-sulfonate in **13** D_2O.

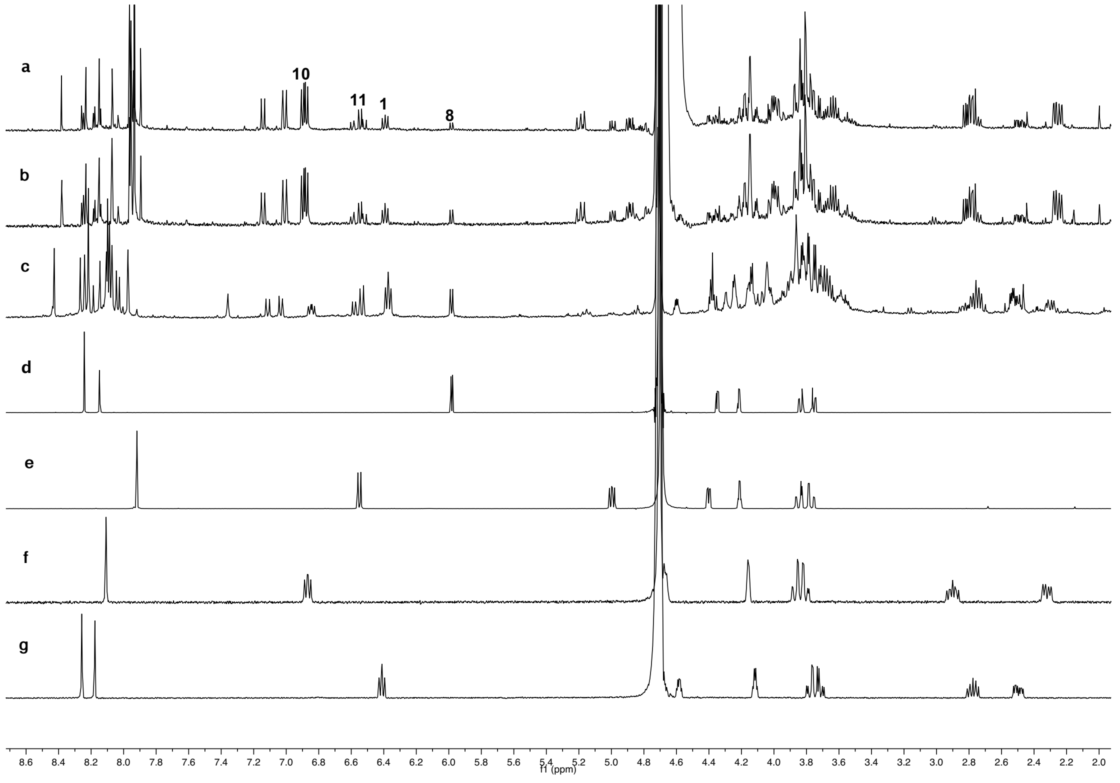

Fig. S25 ^{1}H NMR spectra of the photoreaction of thioanhydroadenosine **6** with sulfite at pH 9, the spiking experiment with adenosine, and spectra of authentic standards. a) ^{1}H NMR spectrum for the reaction mixture after 5 hours of irradiation; b) as a), spiked with adenosine **8**; c) ^{1}H NMR spectrum for the reaction mixture after 24 hours of irradiation; d) ^{1}H NMR spectrum of adenosine **8**; e) ^{1}H NMR spectrum of 8-mercaptoadenosine **11**; f) ^{1}H NMR spectrum of 8-mercaptodeoxyadenosine **10**; and g) ^{1}H NMR spectrum of deoxyadenosine **1**.

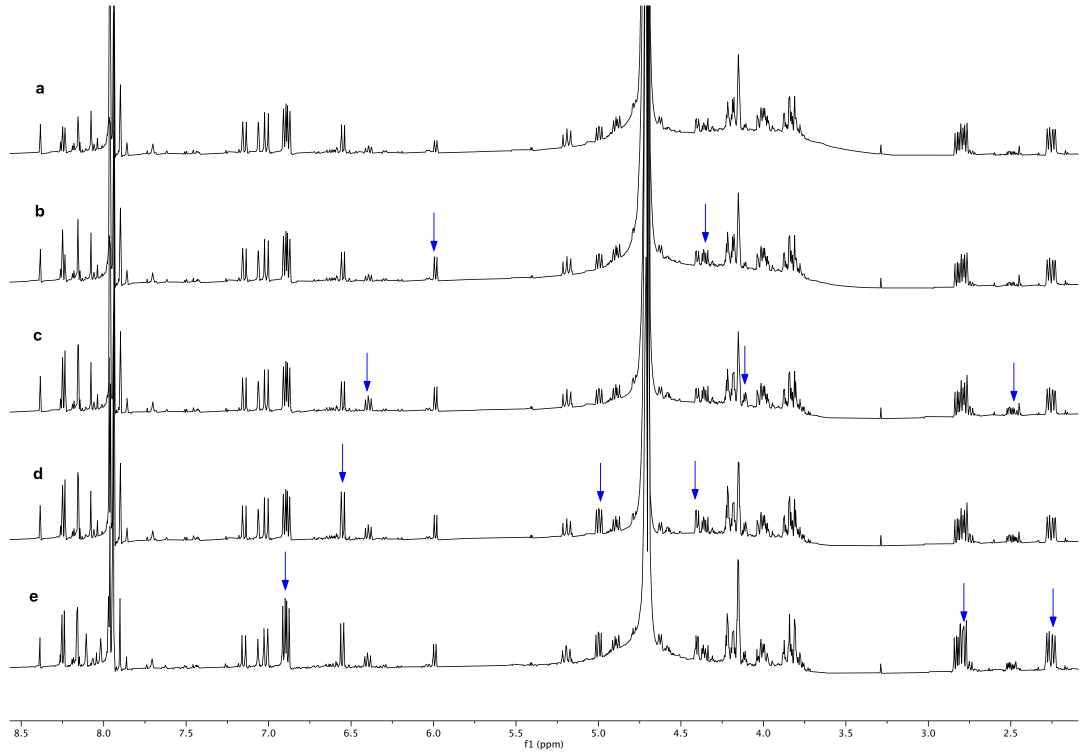

Fig. S26 ^{1}H NMR spectra of the spiking experiments for photoreaction of thioanhydroadenosine **6** with sulfite at pH 9. a) ^{1}H NMR spectrum for the reaction mixture after 2 hours of irradiation; b) as a), spiked with adenosine **8**; c) as b), spiked with deoxyadenosine **1**; d) as c), spiked with 8-mercaptoadenosine **11**; e) as d) spiked with 8-mercaptodeoxyadenosine **10**. Signals from the spiking compounds are indicated with blue arrows, as shown above.

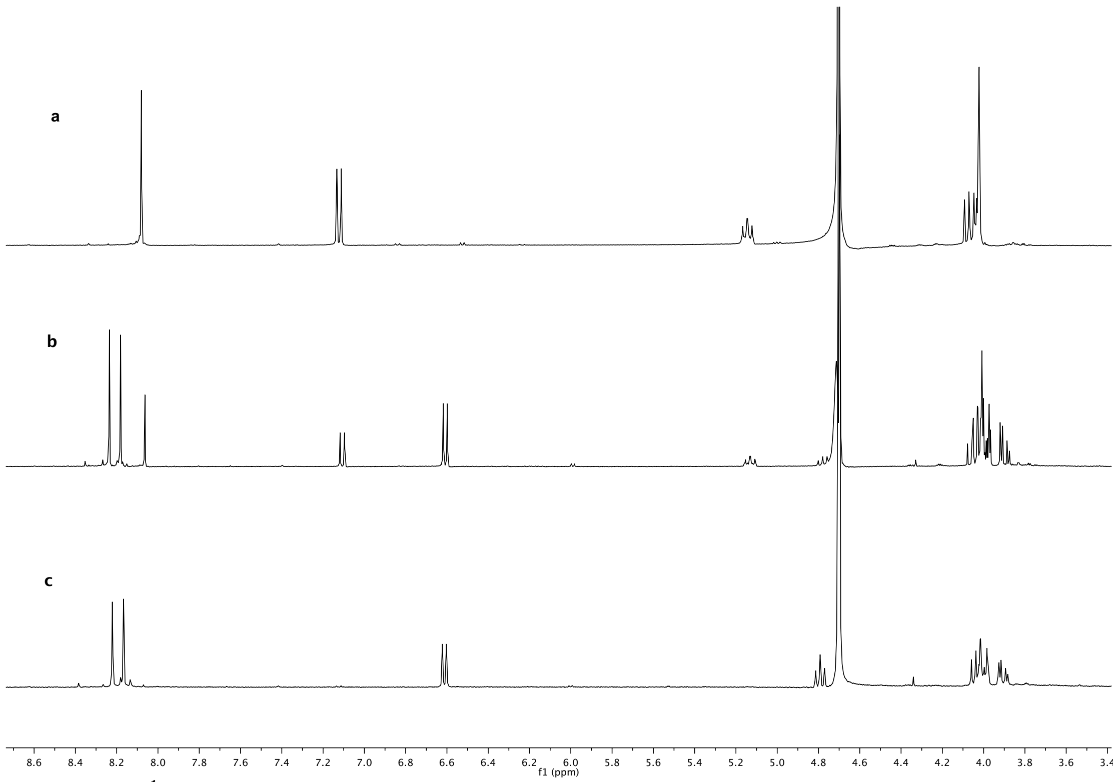

Fig. S27 ^1H NMR spectra for the photoreaction of 8-mercaptoadenosine-2'-β-sulfonate **15**. a) ^1H NMR spectrum of 8-mercaptoadenosine-2'-β-sulfonate **15** before irradiation; b) ^1H NMR spectrum for the mixture after 12 hours of irradiation; c) ^1H NMR spectrum showing full conversion of 8-mercaptoadenosine-2'-β-sulfonate **15** to adenosine-2'-β-sulfonate **13** after 24 hours of irradiation.

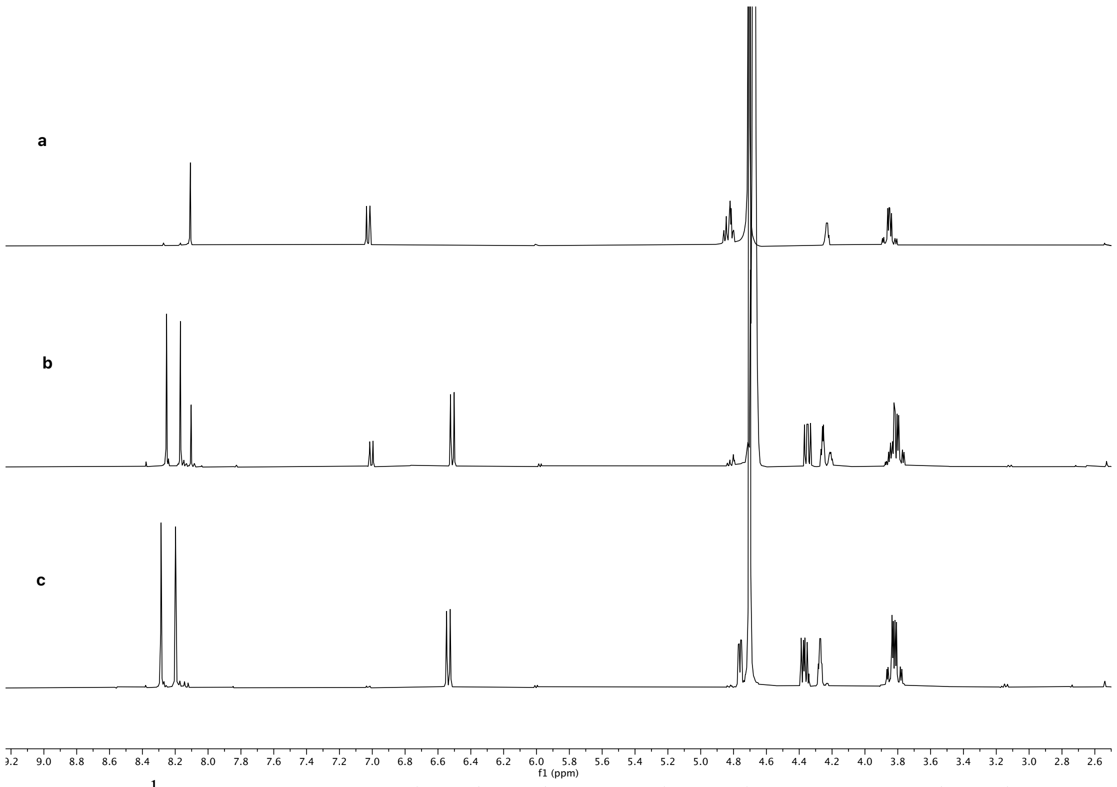

Fig. S28 ^{1}H NMR spectra for the photoreaction of 8-mercaptoadenosine-2'-α-sulfonate **14**. a) ^{1}H NMR spectrum of 8-mercaptoadenosine-2'-α-sulfonate **14** before irradiation; b) ^{1}H NMR spectrum for the mixture after 12 hours of irradiation; c) ^{1}H NMR spectrum showing full conversion of 8-mercaptoadenosine-2'-α-sulfonate **14** to adenosine-2'-α-sulfonate **12** after 24 hours of irradiation.

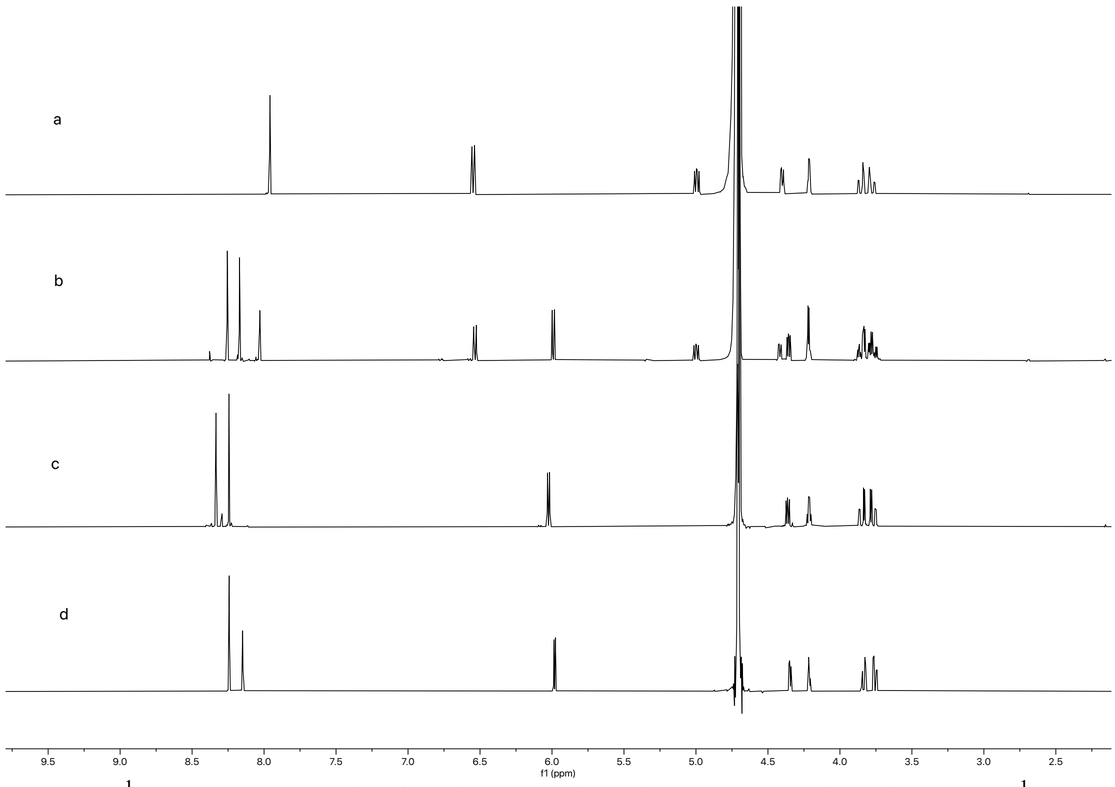

Fig. S29 ^{1}H NMR spectra for the photoreaction of 8-mercaptoadenosine **11**. a) ^{1}H NMR spectrum of 8-mercaptoadenosine **11** before irradiation; b) ^{1}H NMR spectrum for the reaction mixture after 4 hours of irradiation; c) ^{1}H NMR spectrum for the reaction mixture after 12 hours of irradiation; d) ^{1}H NMR spectrum of adenosine **8**.

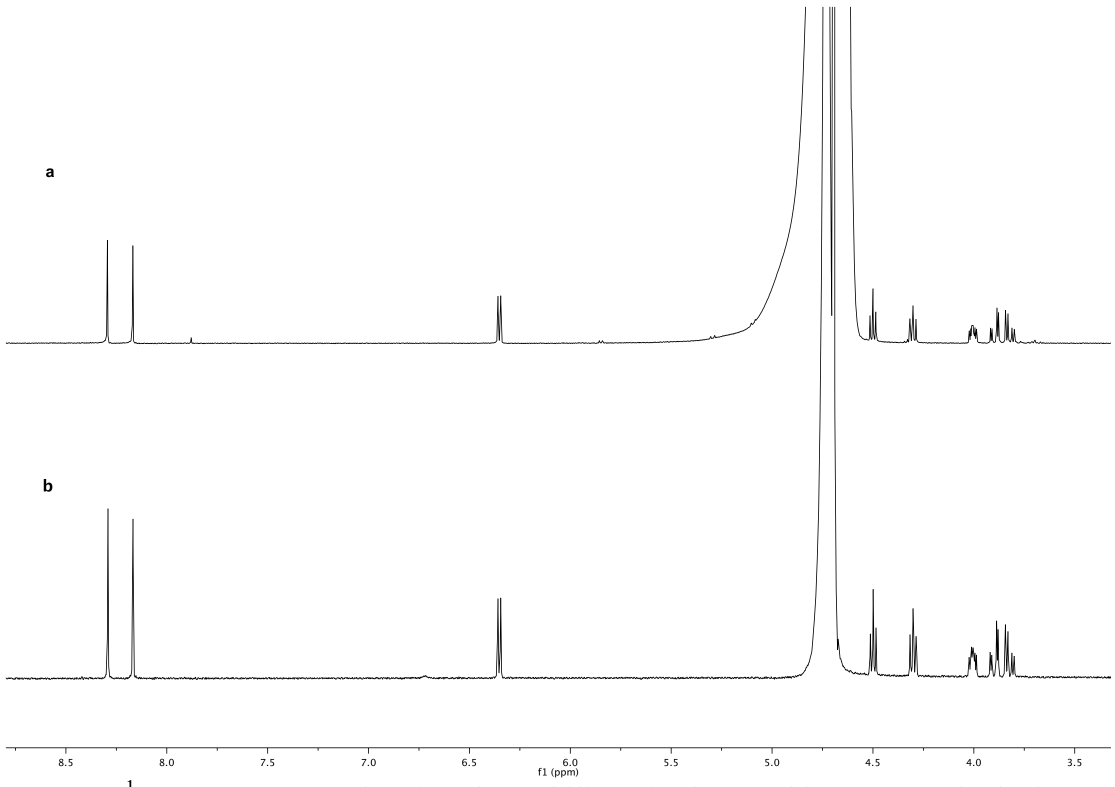

Fig. S30 ^{1}H NMR spectra showing the stability of 9-β-D-arabinofuranosyl adenine **22** towards irradiation with or without sulfite. a) ^{1}H NMR spectrum after 2 h of irradiation with sulfite; b) ^{1}H NMR spectrum after 2 h of irradiation without sulfite.

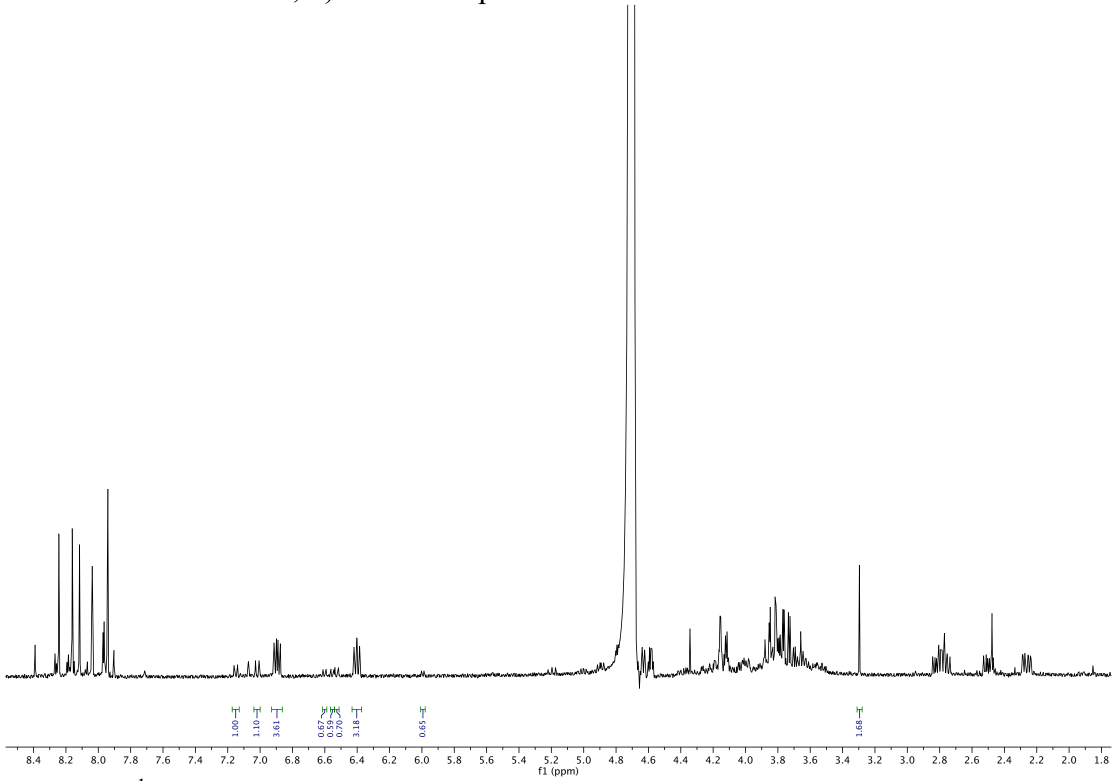

Fig. S31 ^{1}H NMR spectrum of the photoreaction of thioanhydroadenosine **6** with sulfite at pH 8 with added pentaerythritol (5 uL of 25 mM solution).

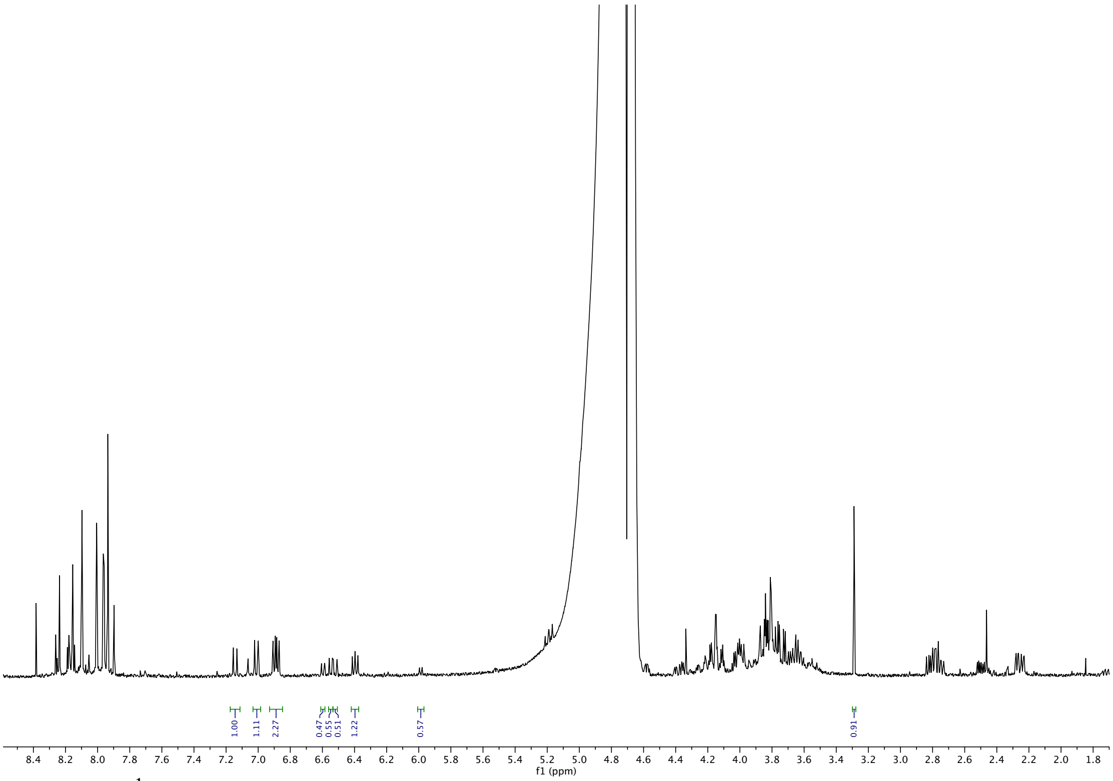

Fig. S32 ^{1}H NMR spectrum of the photoreaction of thioanhydroadenosine **6** with sulfite at pH 9 with added pentaerythritol (4 uL of 25 mM solution).

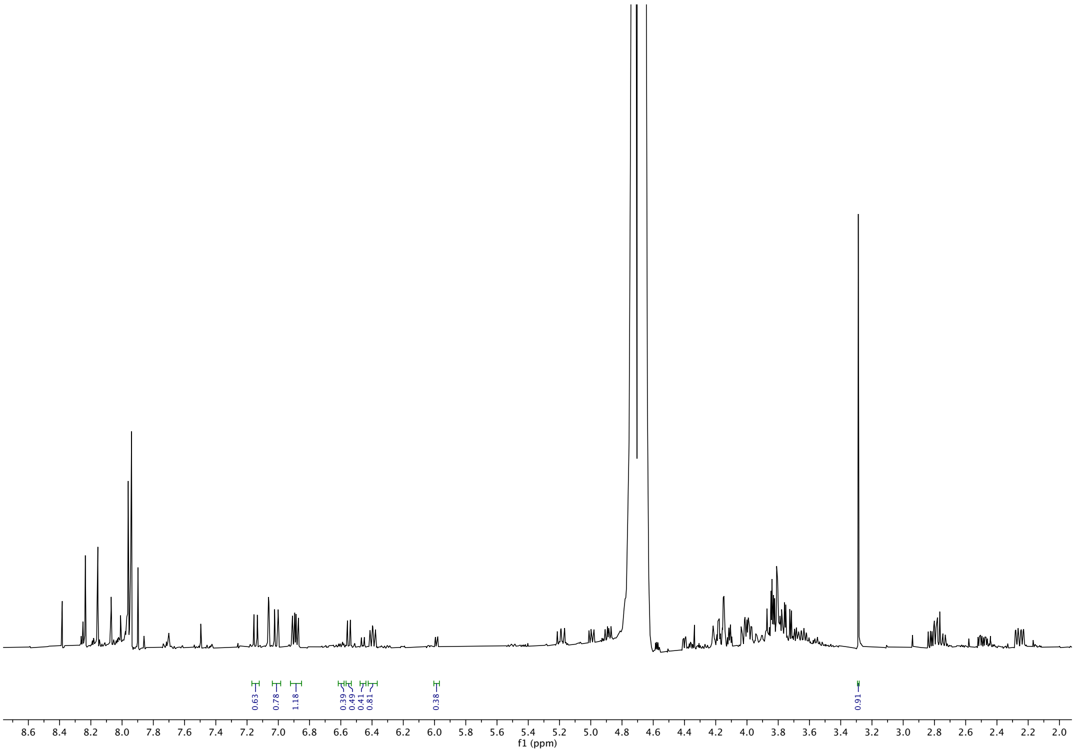

Fig. S33 ^{1}H NMR spectrum of the photoreaction of thioanhydroadenosine **6** with sulfite at pH 10 with added pentaerythritol (5 uL of 25 mM solution).

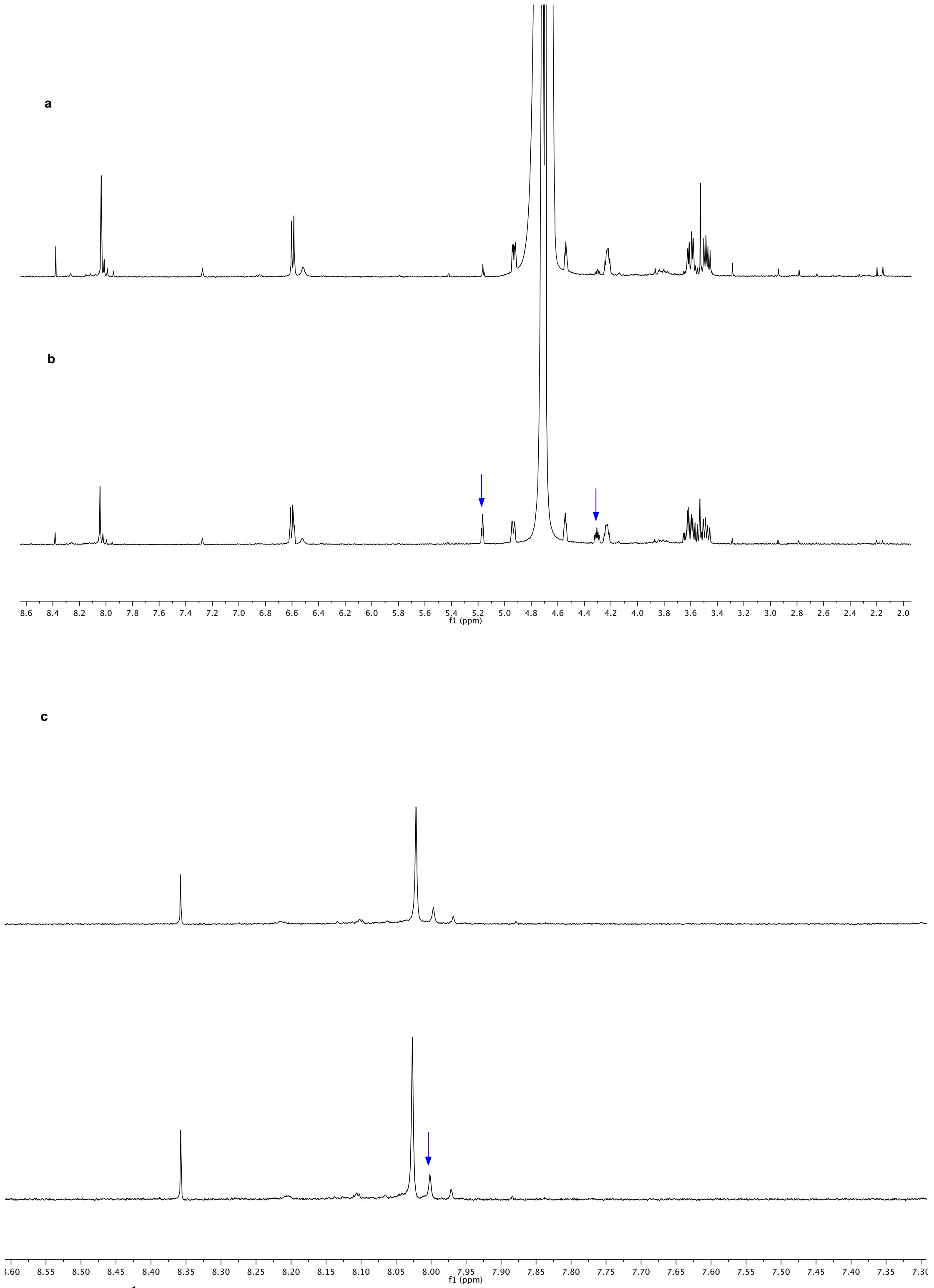

Fig. S34 ^{1}H NMR spectra of the spiking experiment for photoreaction of thioanhydroadenosine **6** without sulfite at pH 9. a) ^{1}H NMR spectrum for the reaction mixture after 2 hours of irradiation; b) as a), spiked with ribal **16**; c) Enlarged region of ^{1}H NMR spectra for the spiking experiment with 8-mercaptoadenine **17**. Signals from the spiking compounds are indicated with blue arrows, as shown above.

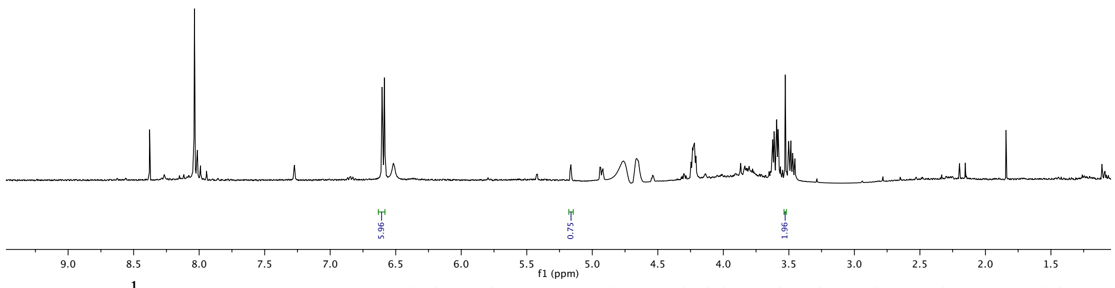

Fig. S35 ^{1}H NMR spectrum of the photoreaction of thioanhydroadenosine **6** without sulfite at pH 9 with added pentaerythritol (10 uL of 25 mM solution).

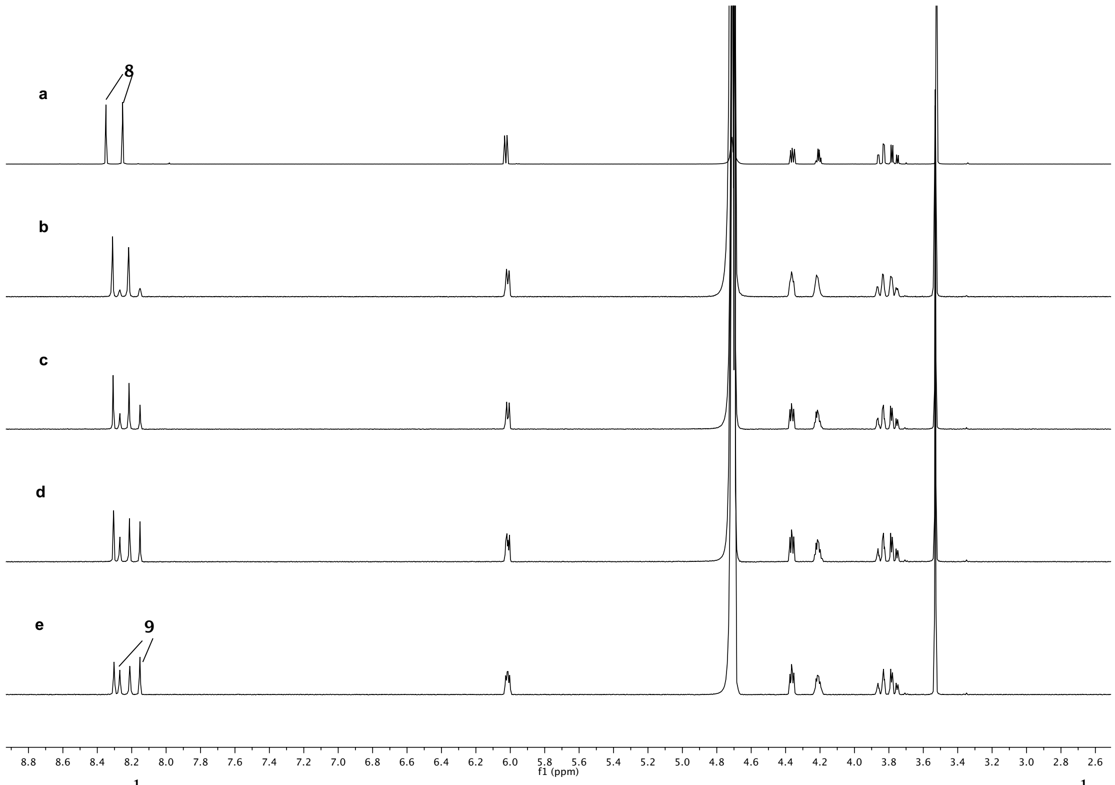

Fig. S36 ^{1}H NMR spectra for the reactions of adenosine **8** with nitrous acid. a) ^{1}H NMR spectrum of adenosine **8**; b) ^{1}H NMR spectrum of the reaction mixture of adenosine **8** with nitrous acid after 1 day; c) ^{1}H NMR spectrum of the reaction mixture of adenosine **8** with nitrous acid after 2 days; d) ^{1}H NMR spectrum of the reaction mixture of adenosine **8** with nitrous acid after 3 day; e) ^{1}H NMR spectrum of

the reaction mixture of adenosine **8** with nitrous acid after 4 day (ratio of **8**:**9** is 51:49).

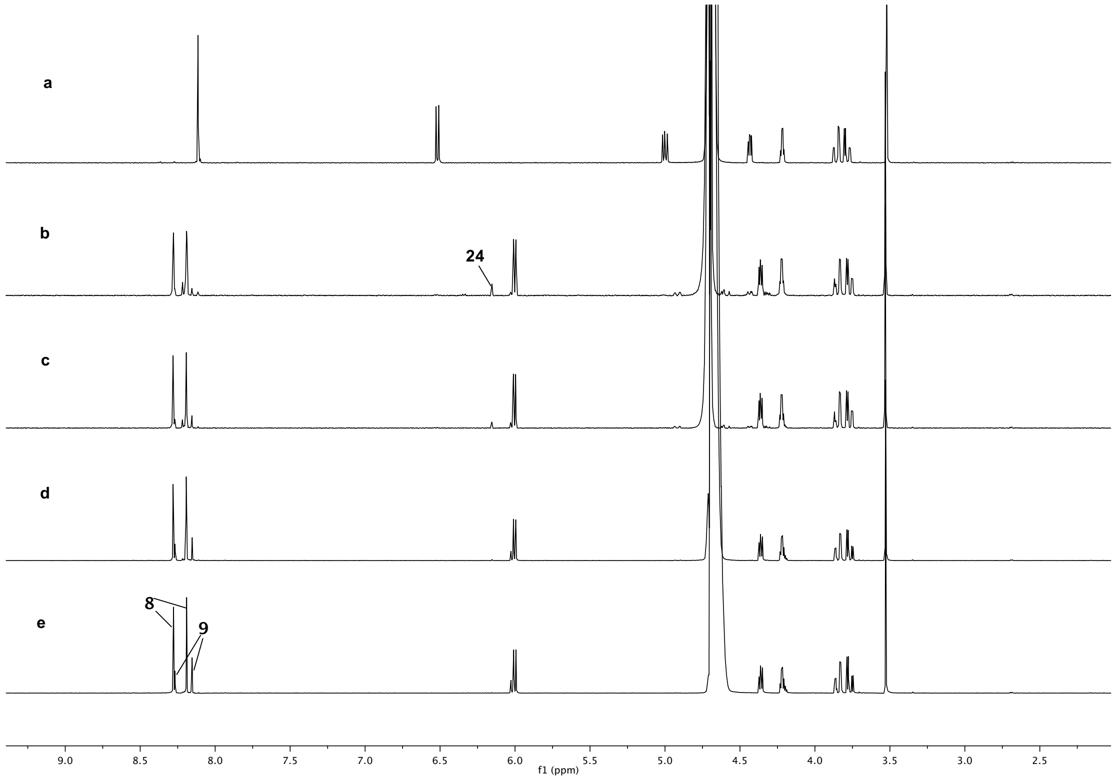

Fig. S37 ^{1}H NMR spectra for the reactions of 8-mercaptoadenosine **11** with nitrous acid. a) ^{1}H NMR spectrum of 8-mercaptoadenosine **11**; b) ^{1}H NMR spectrum of the reaction mixture of 8-mercaptoadenosine **11** with nitrous acid after 2 days; c) ^{1}H NMR spectrum of the reaction mixture of 8-mercaptoadenosine **11** with nitrous acid after 4 days; d) ^{1}H NMR spectrum of the reaction mixture of 8-mercaptoadenosine **11** with nitrous acid after 8 days; e) ^{1}H NMR spectrum of the reaction mixture of 8-mercaptoadenosine **11** with nitrous acid after 12 days (ratio of **8**:**9** is 76:24).

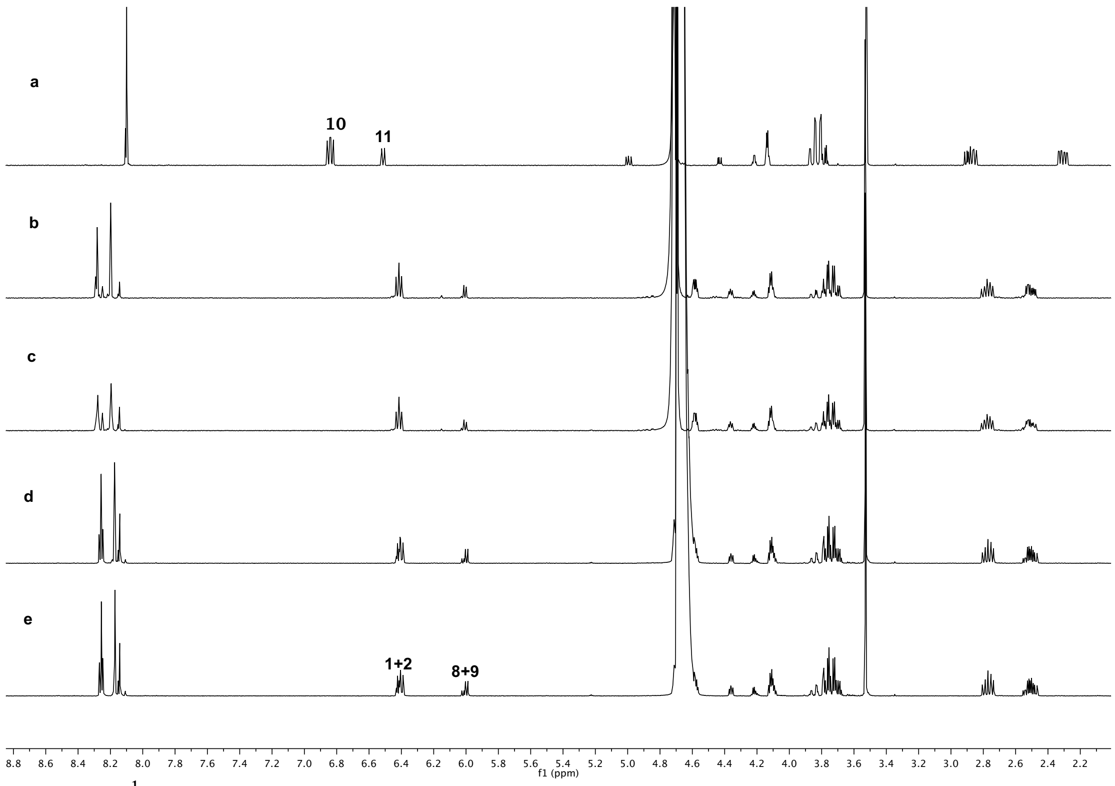

Fig. S38 ^{1}H NMR spectra for the reactions of 8-mercaptodeoxyadenosine **10** and 8-mercaptoadenosine **11** with nitrous acid. a) ^{1}H NMR spectrum of the mixture of 8-mercaptodeoxyadenosine **10** and 8-mercaptoadenosine **11** (ratio of **10**:**11** is 3:1); b) ^{1}H NMR spectrum of the reaction mixture of 8-mercaptodeoxyadenosine **10** and 8-mercaptoadenosine **11** with nitrous acid after 2 days; c) b) ^{1}H NMR spectrum of the reaction mixture of 8-mercaptodeoxyadenosine **10** and 8-mercaptoadenosine **11** with nitrous acid after 4 days; d) b) ^{1}H NMR spectrum of the reaction mixture of 8-mercaptodeoxyadenosine **10** and 8-mercaptoadenosine **11** with nitrous acid after 8 days; e) b) ^{1}H NMR spectrum of the reaction mixture of 8-mercaptodeoxyadenosine **10** and 8-mercaptoadenosine **11** with nitrous acid after 12 days (ratio of **1**:**2**:**8**:**9** is 49:27:17:7).

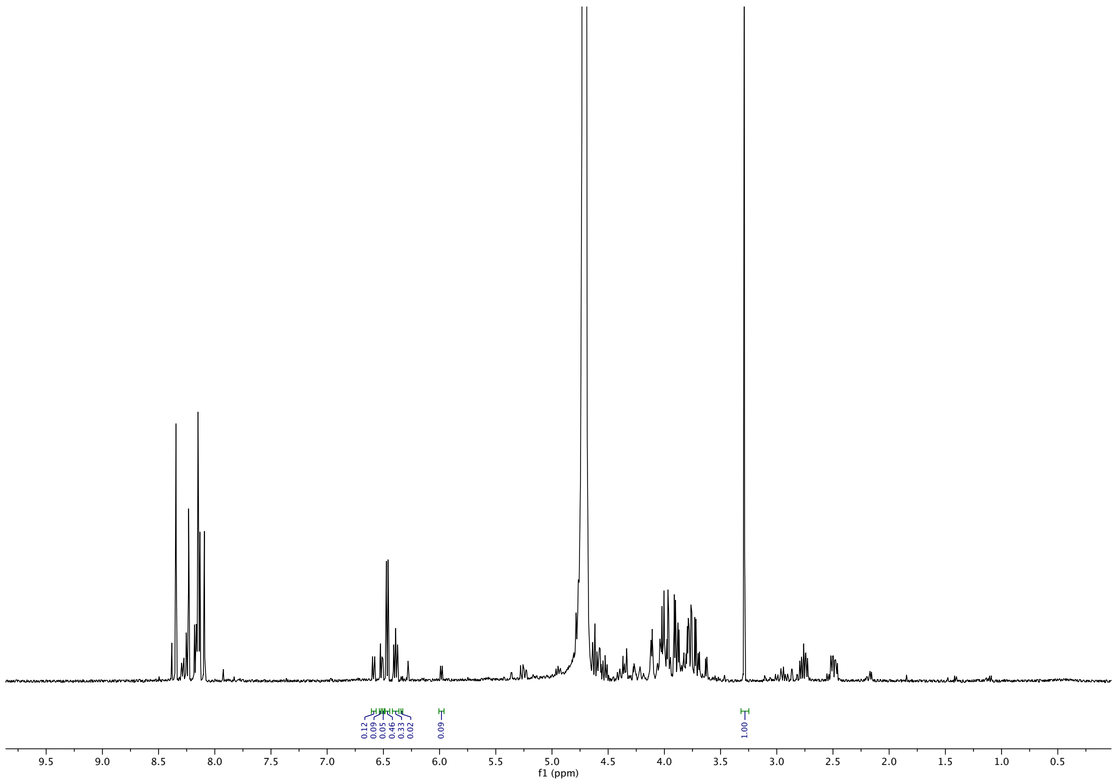

Fig. S39 ^{1}H NMR spectrum of the photoreaction of 2'-deoxy-β-bromoadenosine **23** with sulfite at pH 9 with added pentaerythritol (25 uL of 25 mM solution).

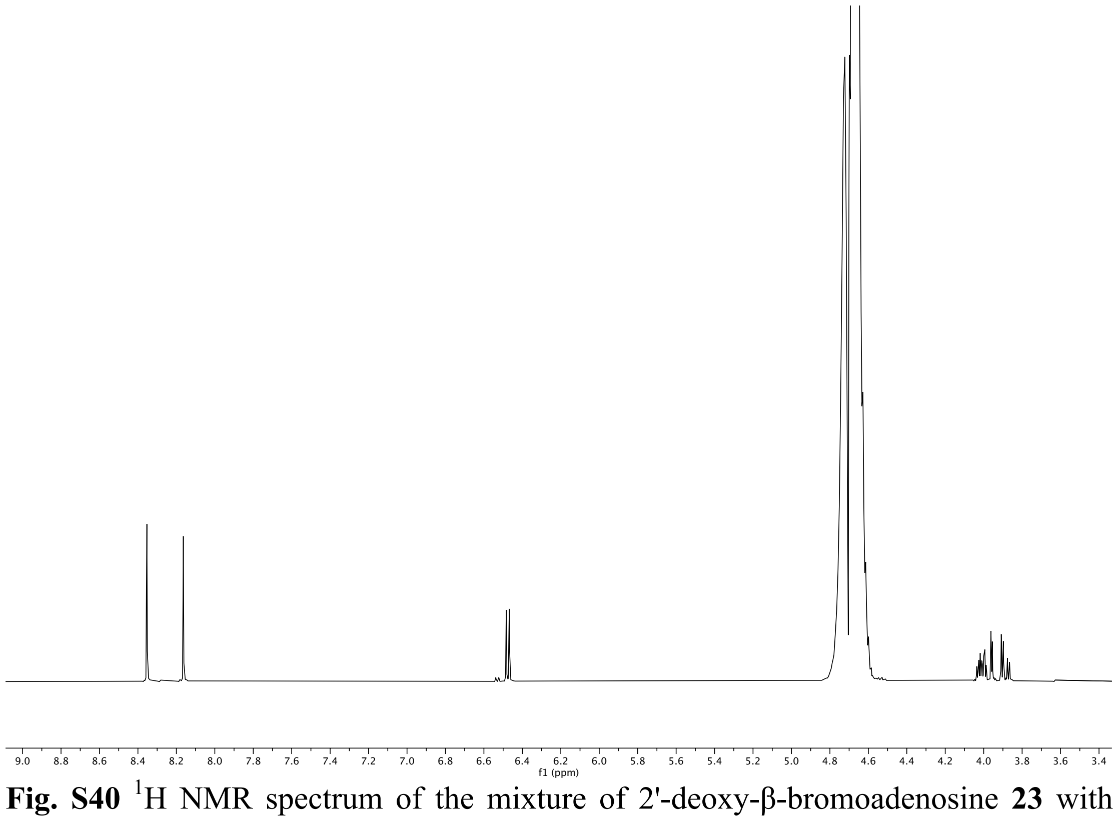

Fig. S40 ^{1}H NMR spectrum of the mixture of 2'-deoxy-β-bromoadenosine **23** with sulfite at pH 9 after 3 hours in the dark.

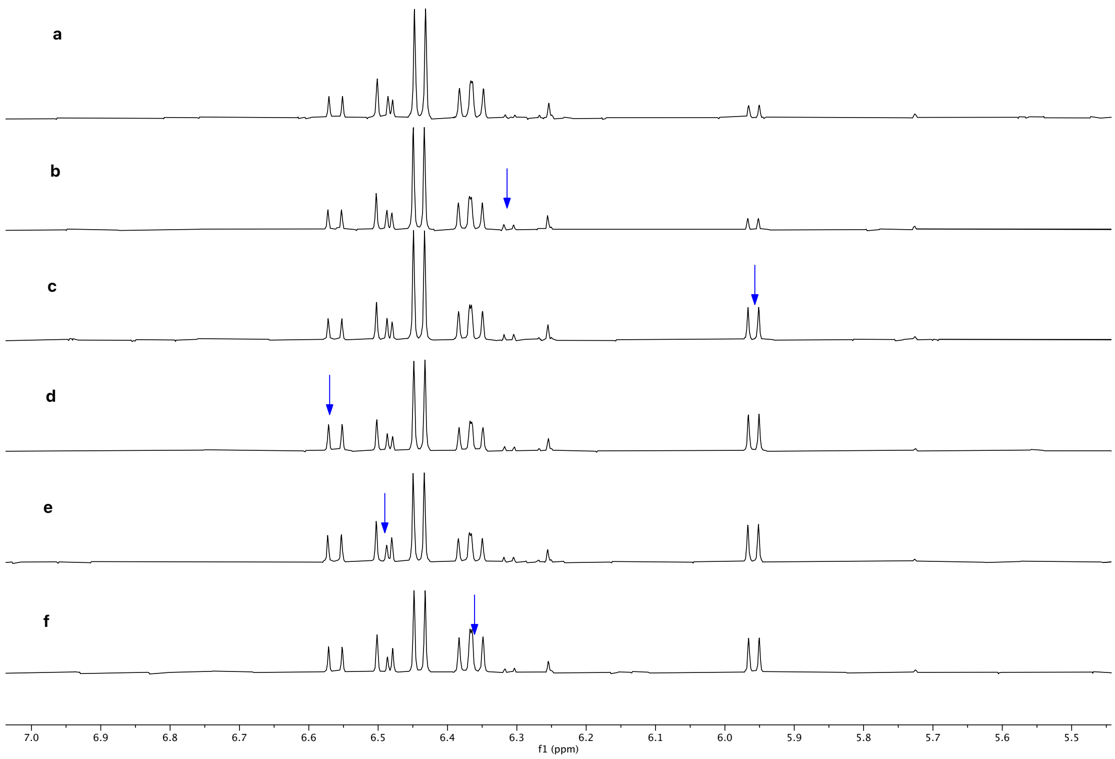

Fig. S41 ^{1}H NMR spectra (1' proton region) of the spiking experiments for photoreaction of 2'-deoxy-β-bromoadenosine **23** with sulfite at pH 9. a) ^{1}H NMR spectrum for the reaction mixture after 3 hours of irradiation; b) as a), spiked with arabino-adenosine **22**; c) as b), spiked with adenosine **8**; d) as c), spiked with adenosine-2'-α-sulfonate **12**; e) as d) spiked with adenosine-2'-β-sulfonate **13**; f) as e), spiked with deoxyadenosine **1**. Signals from the spiking compounds are indicated with blue arrows, as shown above.

Rxn 1 (no labelling, pH 11 control)

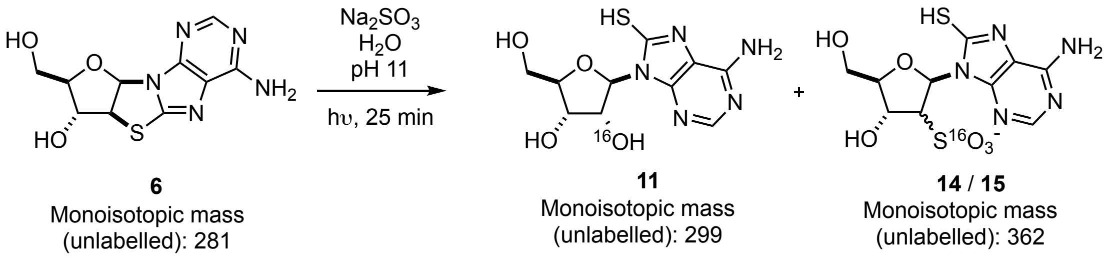

UV (260 nm) of LCMS for Rxn 1:

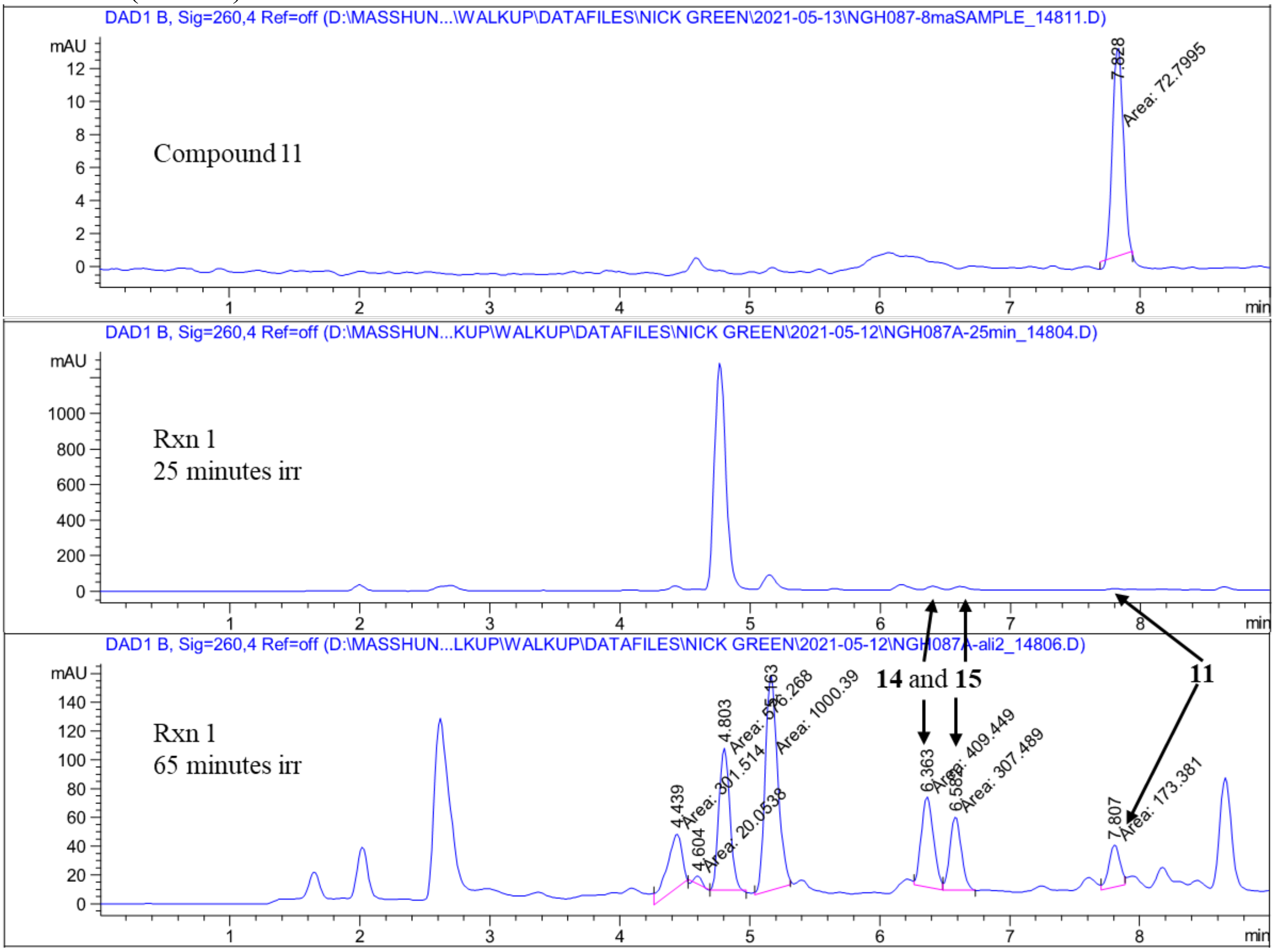

Mass Spec (ESI, negative) of annotated peaks above, at 25 minutes:

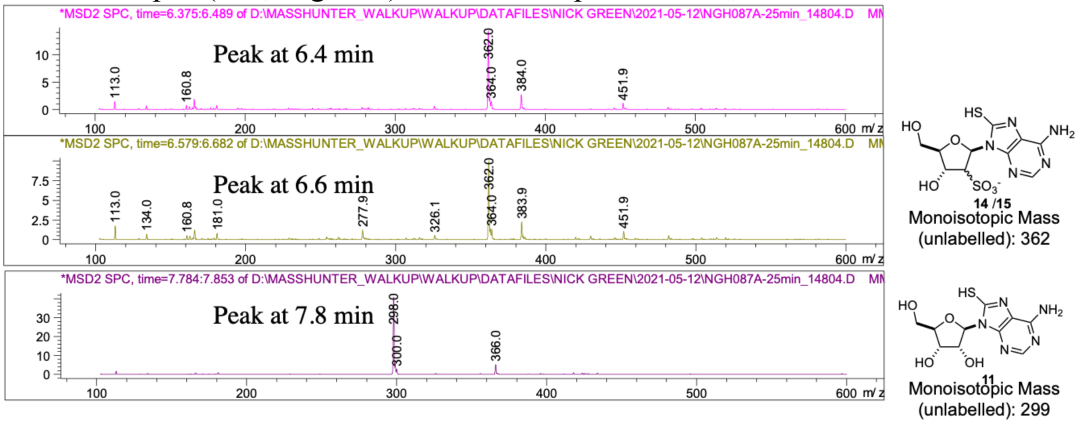

Fig. S42 LCMS data for control experiment with **6** (Rxn1).

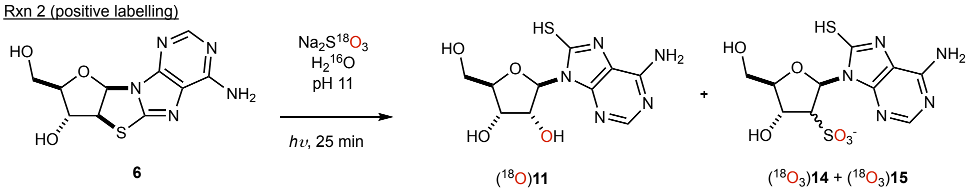

Chromatogram from LCMS after 25 min:

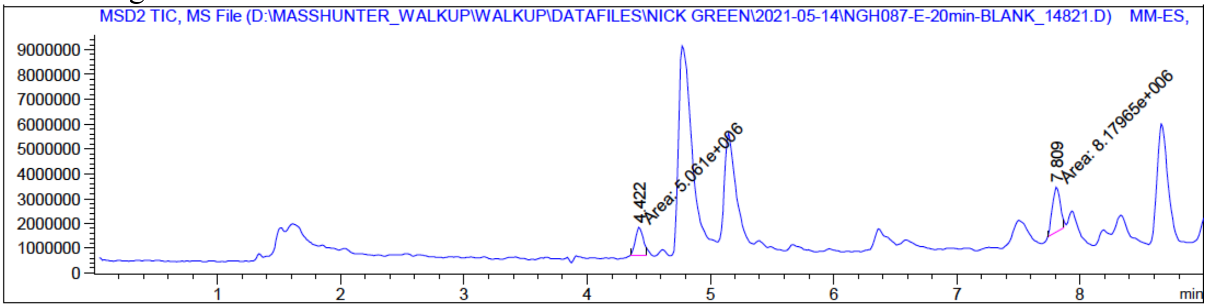

Mass spec (ESI, negative) extraction of peaks at 6.4 min and 7.8 min:

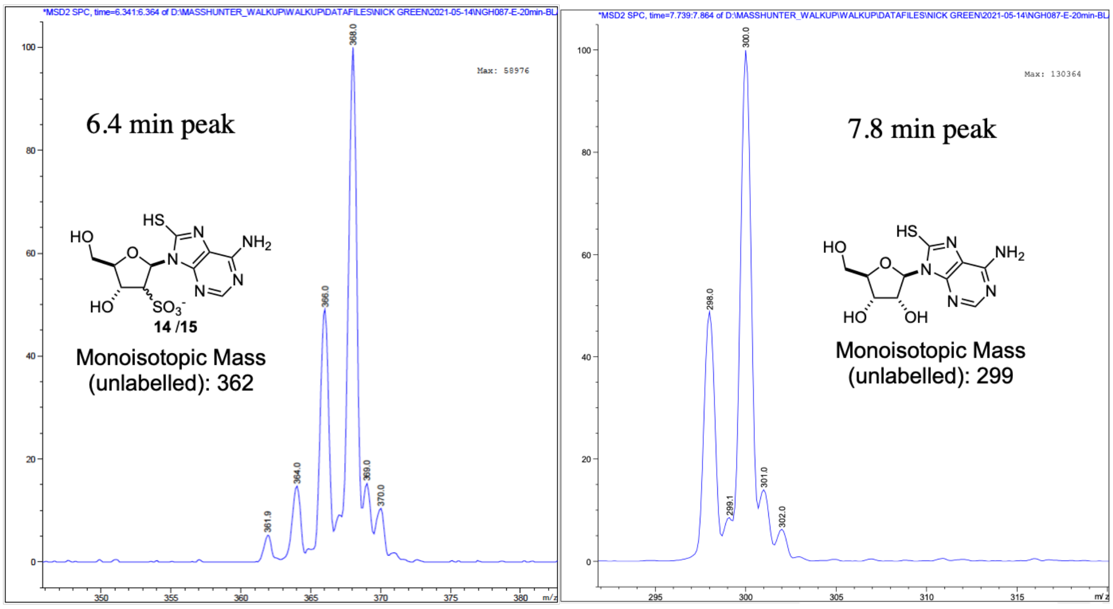

Extensive labelling observed for both compounds.

Fig. S43 LCMS data for positive labelling experiment with **6** (Rxn2).

Rxn 3 (negative labelling)

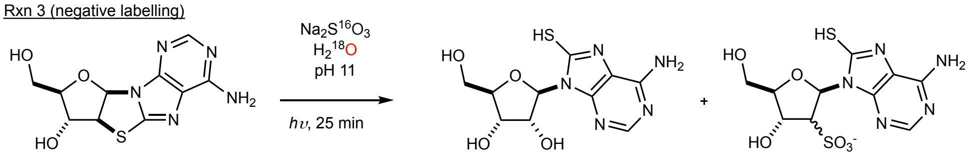

6 (^{16}O)**11** $(^{16}O_3)$**14** + $(^{16}O_3)$**15**

Chromatogram from LCMS after 25 min:

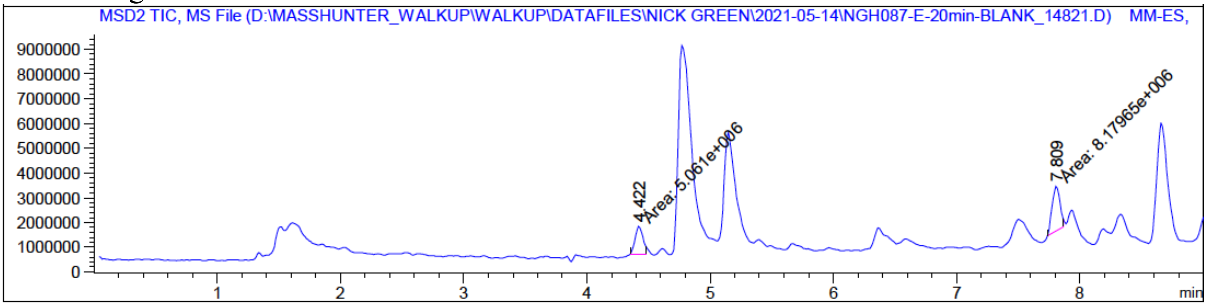

Mass spec (ESI, negative) extraction of peaks at 6.4 min and 7.8 min:

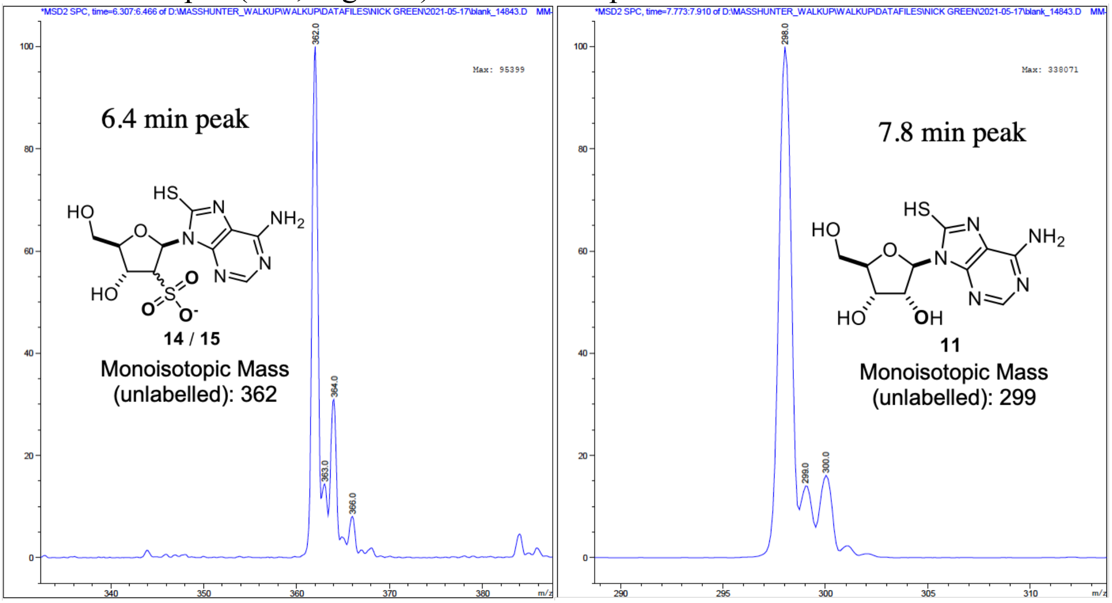

Minimal labelling observed for both compounds.

Fig. S44 LCMS data for negative labelling experiment with **6** (Rxn3).

Rxn 4 (no labelling, pH 11 control)

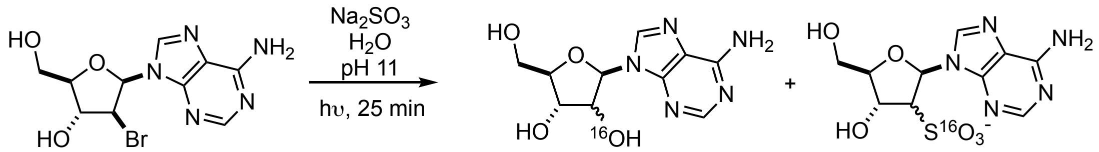

23 Monoisotopic mass (unlabelled): 329

8, 22 Monoisotopic mass (unlabelled): 267

12, 13 Monoisotopic mass (unlabelled): 330

Chromatogram from LCMS (UV 260nm):

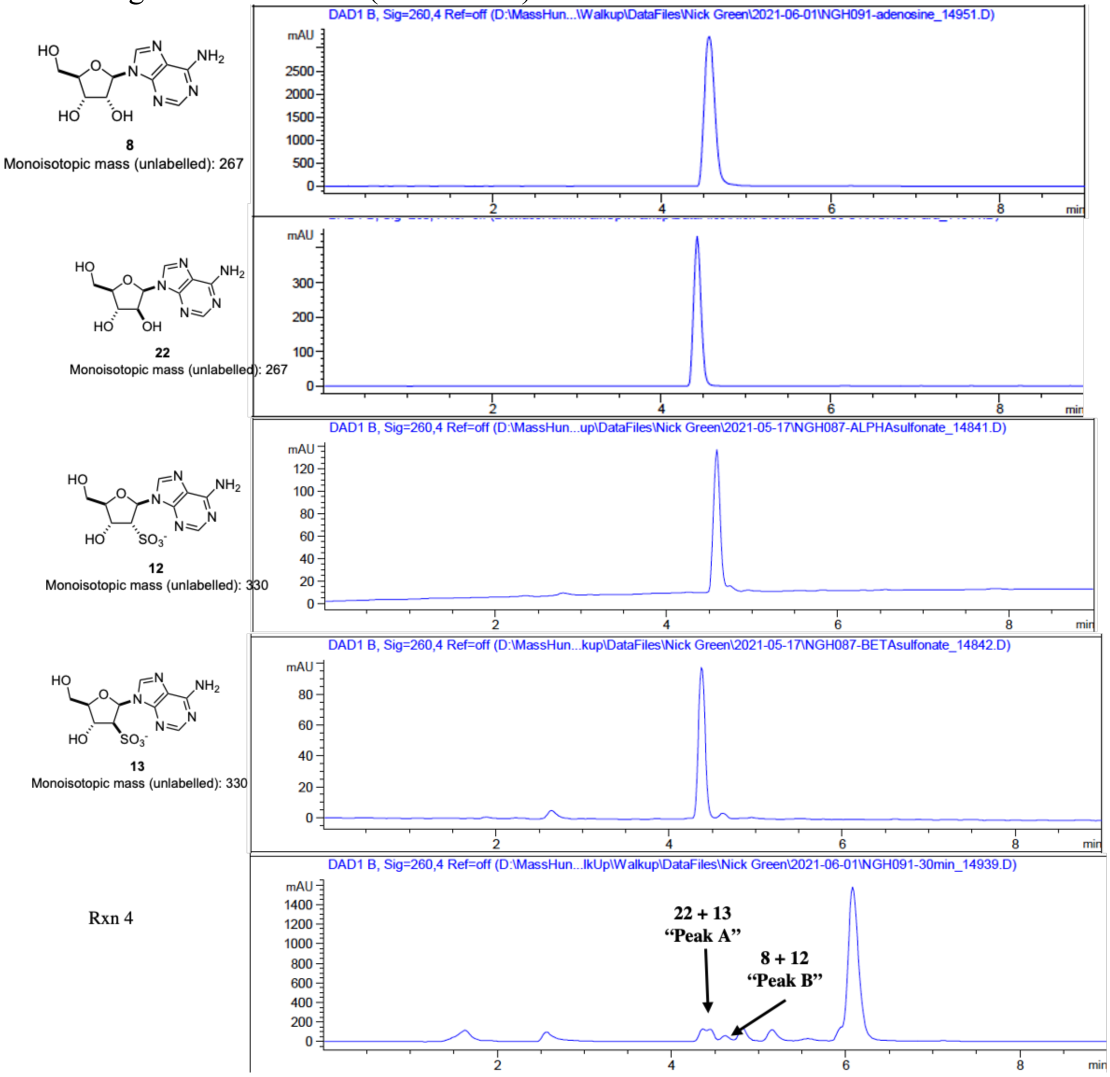

Fig. S45 UV Chromatogram from LCMS for control experiment with **23** (Rxn 4).

Rxn 4 (control)
"Peak A"
ESI-

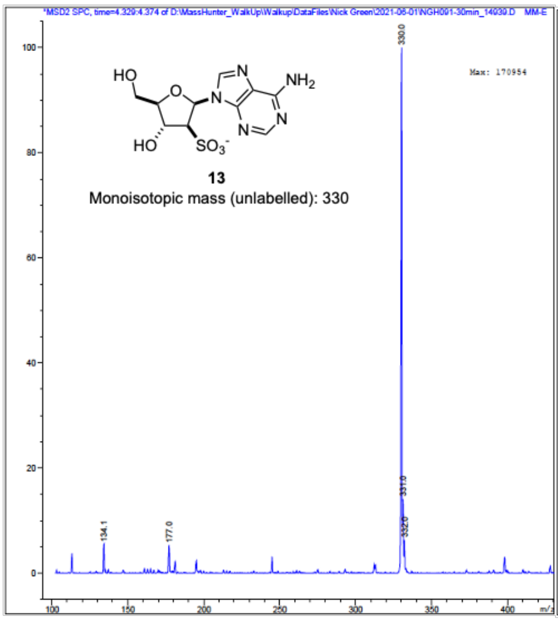

Rxn 4 (control)
"Peak A"
ESI+

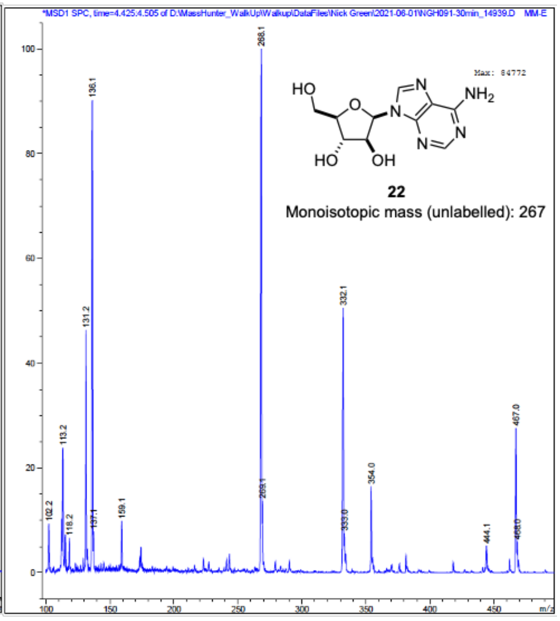

Rxn 4 (control)
"Peak B"
ESI-

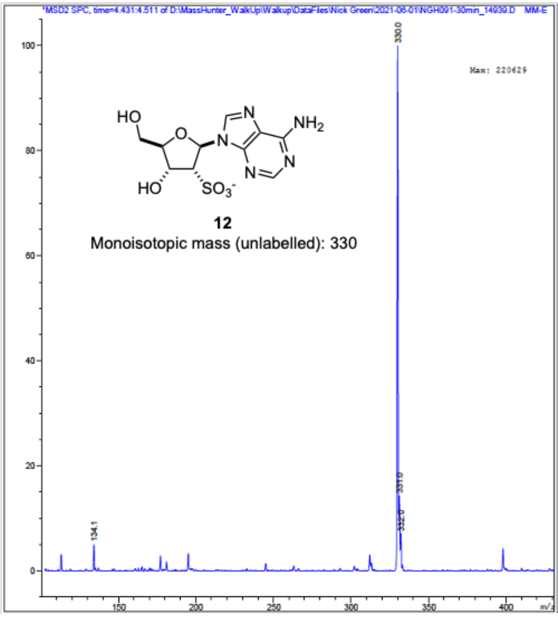

Rxn 4 (control)
"Peak B"
ESI+

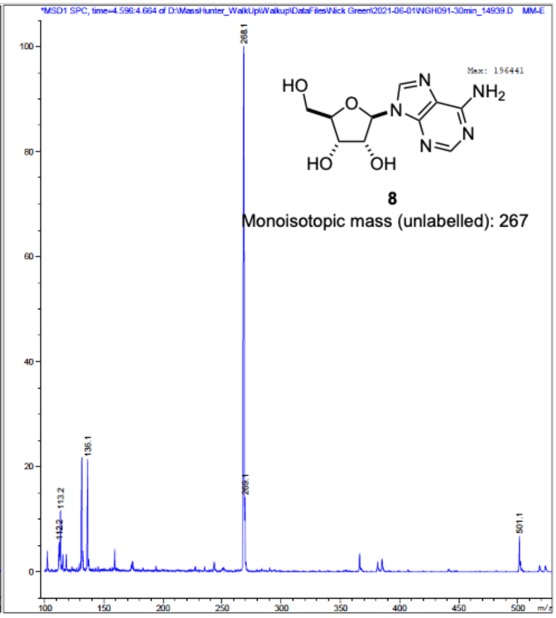

Fig. S46 ESI-MS (positive and negative) of key peaks from control experiment with **23** (Rxn 4).

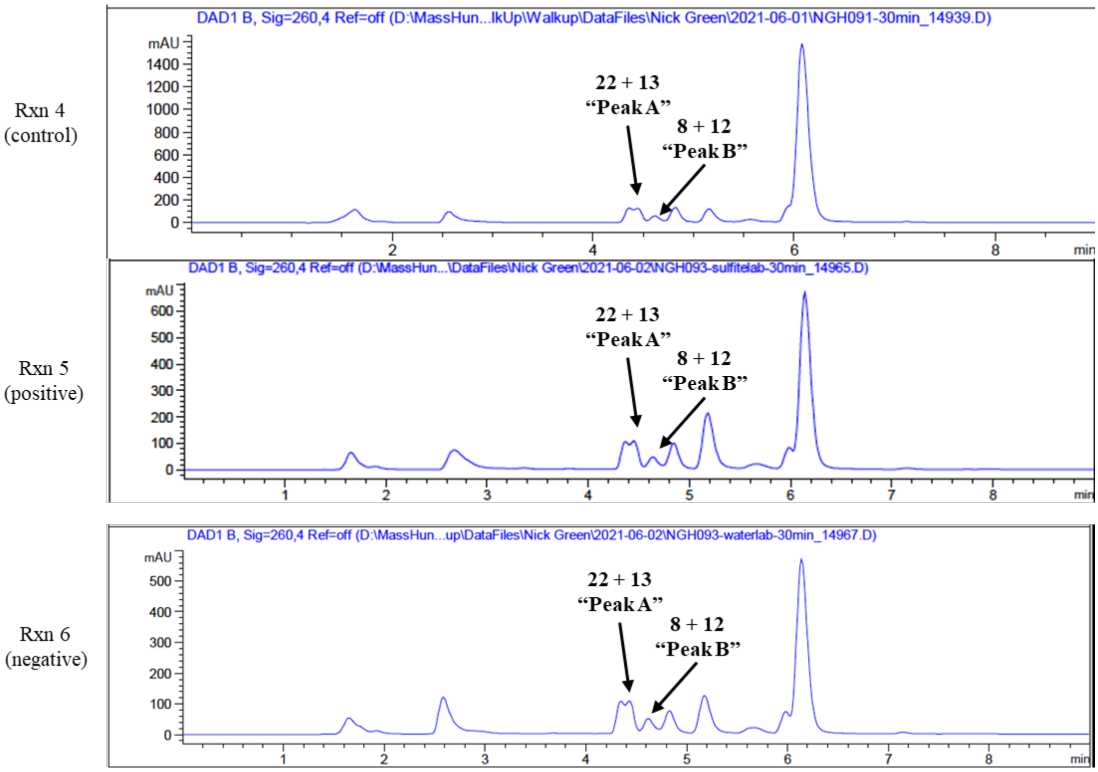

Fig. S47 Comparison of control reaction with positive and negative labelling experiments with compound **23**.

Rxn 5 (positive)
"Peak A"
ESI-

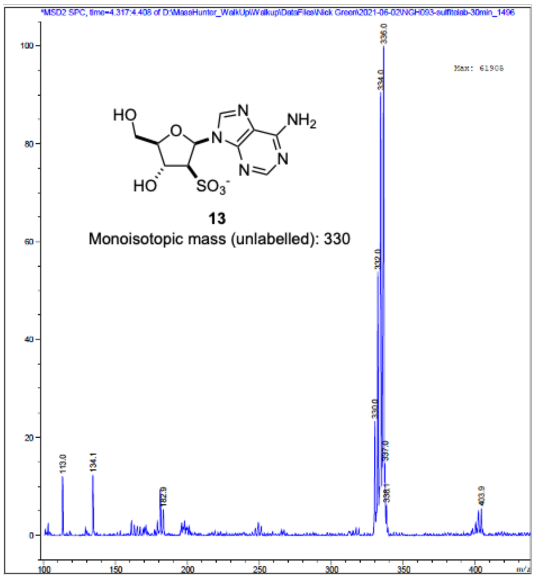

Rxn 5 (positive)
"Peak A"
ESI+

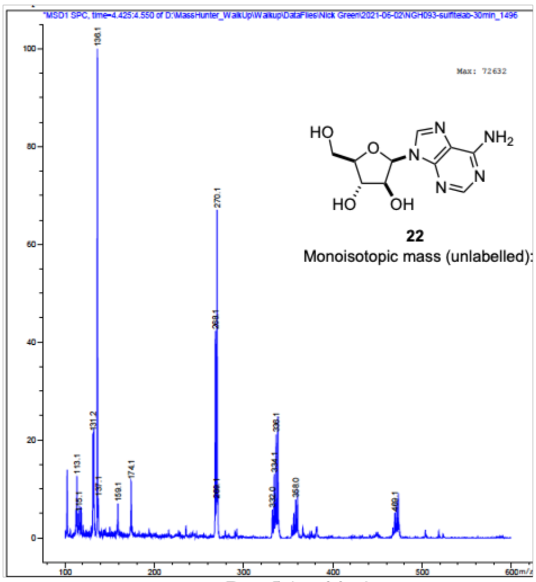

Rxn 5 (positive)
"Peak B"
ESI-

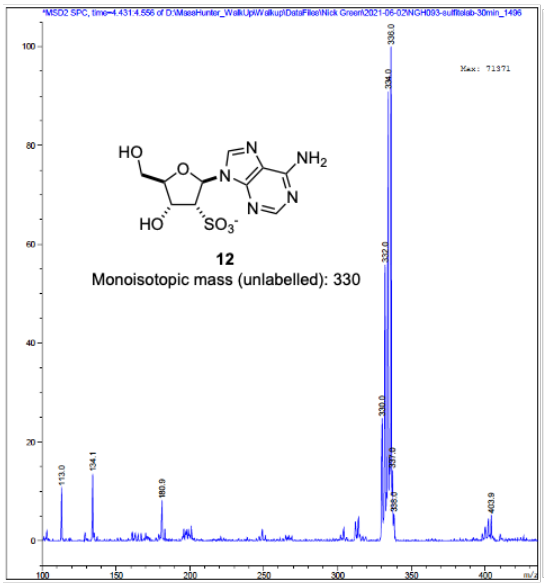

Rxn 5 (positive)
"Peak B"
ESI+

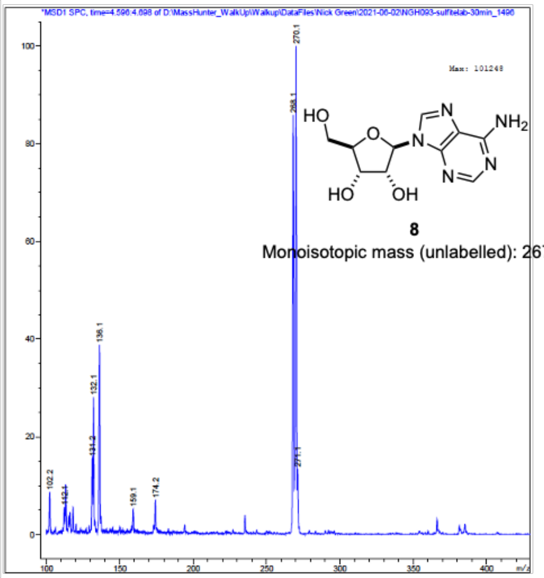

Fig. S48 ESI-MS (positive and negative) of key peaks from positive labelling experiment with **23** (Rxn 5).

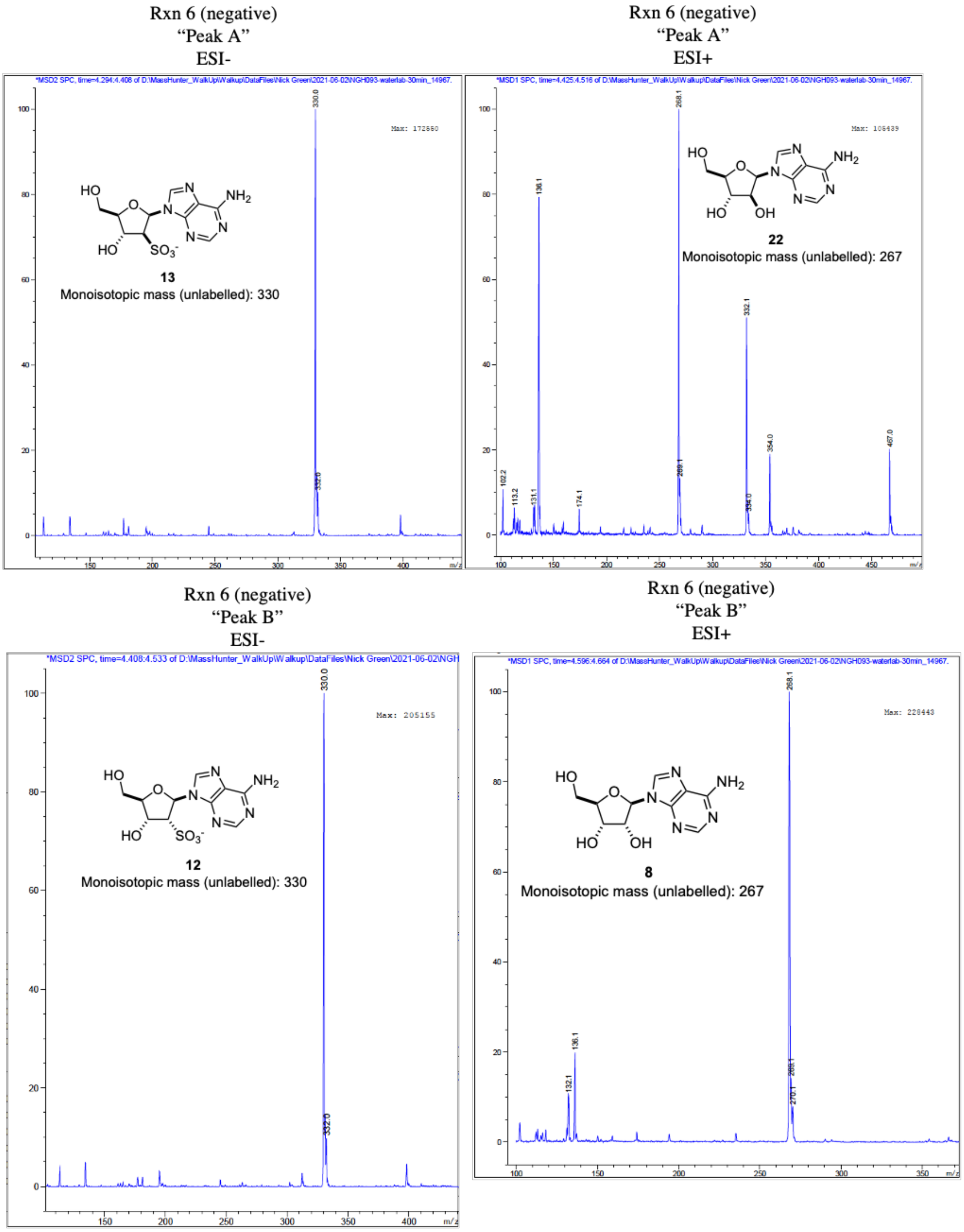

Fig. S49 ESI-MS (positive and negative) of key peaks from negative labelling experiment with **23** (Rxn 6).

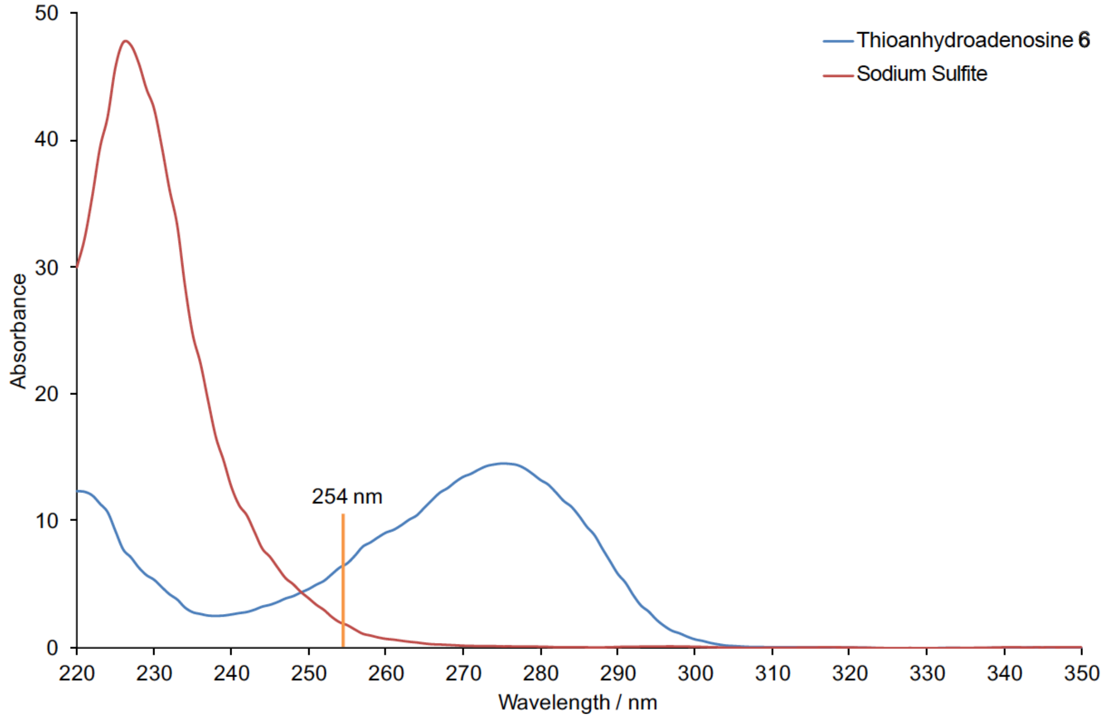

Fig. S50 *UV-Vis* spectra of **6** (1 mM at pH 9, in blue) and sulfite (100 mM at pH 9, in red)

References:

1. Lefoix, M.; Mathis, G.; Kleinmann, T.; Truffert, J.-C.; Asseline, U. Pyrazolo[1,5-a]-1,3,5-triazine C-Nucleoside as Deoxyadenosine Analogue: Synthesis, Pairing, and Resistance to Hydrolysis. *J. Org. Chem.* **79**, 3221-3227 (2014).
2. Stolarski, R; Hagberg, C.-E.; Shugar, D. *Eur. J. Biochem.* Studies on the dynamic syn-anti equilibrium in purine nucleosides and nucleotides with the aid of ^{1}H and ^{13}C NMR spectroscopy. **138**, 187-192 (1984).
3. Betts, R. H.; Voss, R. H. The kinetics of oxygen exchange between the sulfite ion and water. *Can. J. Chem.* **48**, 2035-2041(1970).
